# Development of peptides targeting receptor binding site of the envelope glycoprotein to contain the West Nile virus infection

**DOI:** 10.1038/s41598-021-99696-w

**Published:** 2021-10-11

**Authors:** Patrícia Mertinková, Evelína Mochnáčová, Katarína Bhide, Amod Kulkarni, Zuzana Tkáčová, Jana Hruškovicová, Mangesh Bhide

**Affiliations:** 1 Laboratory of Biomedical Microbiology and Immunology, The University of Veterinary Medicine and Pharmacy in Košice, Komenského 73, 04181 Košice, Slovakia; 2 Institute of Neuroimmunology of Slovak Academy of Sciences, Dubravska cesta 9, 84510 Bratislava, Slovakia

**Keywords:** High-throughput screening, Drug discovery

## Abstract

West Nile virus (WNV), re-emerging neurotropic flavivirus, can cross the blood–brain barrier (BBB) and cause fatal encephalitis and meningitis. Infection of the human brain microvascular endothelial cells (hBMECs), building blocks of the BBB, represents the pivotal step in neuroinvasion. Domain III (DIII) of the envelope (E) glycoprotein is a key receptor-binding domain, thus, it is an attractive target for anti-flavivirus strategies. Here, two combinatorial phage display peptide libraries, Ph.D.-C7C and Ph.D.-12, were panned against receptor-binding site (RBS) on DIII to isolate peptides that could block DIII. From series of pannings, nine peptides (seven 7-mer cyclic and two 12-mer linear) were selected and overexpressed in *E. coli* SHuffle T5. Presence of disulfide bond in 7-mer peptides was confirmed with thiol-reactive maleimide labeling. Except for linear peptide 19 (HYSWSWIAYSPG), all peptides proved to be DIII binders. Among all peptides, 4 cyclic peptides (CTKTDVHFC, CIHSSTRAC, CTYENHRTC, and CLAQSHPLC) showed significant blocking of the interaction between DIII and hBMECs, and ability to neutralize infection in cultured cells. None of these peptides showed toxic or hemolytic activity. Peptides identified in this study may serve as potential candidates for the development of novel antiviral therapeutics against WNV.

## Introduction

West Nile virus (WNV) is a single-stranded, positive-sense RNA virus with tropism towards the nervous system. Since its discovery in the West Nile district of Uganda in 1937^1^, it has spread around the globe and became the most widely distributed mosquito-borne arbovirus^2^ responsible for repetitive outbreaks of febrile illness and meningoencephalitis^3^. Nearly 80% of the infections are subclinical, while in rest of the cases the infection may range from flu-like manifestations (known as West Nile fever) to severe neurological illness resulting in long-term sequelae or death^4^. Although neurotropic WNV causes meningitis, encephalitis, or acute poliomyelitis-like syndrome, which is developed in less than 1% of infected individuals, the fatality rate carries approximately 10%^5^. The highest incidence of the WNV infection (1,605 human cases) was reported in 2018 in Europe, of those cases 61.8% were with neurological symptoms^6^.

WNV reaches the CNS via the hematogenous route by crossing the blood–brain barrier (BBB)^7^, which is a selective barrier made up of brain microvascular endothelial cells (BMECs), pericytes, astrocytes, and the basal lamina^8^. Several routes of WNV entry across the BBB are described, which include (i) cell-free viral infection of BMECs with no direct effect on the barrier integrity^9,10^; (ii) viral entry within infected leukocytes (Trojan horse mechanism)^11,12^; or (iii) viral invasion through the cytokine-mediated BBB breakdown^13,14^, although the relevance of BBB leakage during WNV infection is still controversial^15^. Since BMECs constitute a primary barrier, direct infection of endothelial cells is one of the crucial events that initiates viral transmigration into the CNS. Once infected with WNV, BMECs induces the expression of adhesion molecules, which promotes the transendothelial migration of leukocytes, modulates BBB permeability^16,17^ and assists in the trafficking of WNV-infected immune cells^12^.

Despite a plethora of research in antiviral therapeutics, the development of reliable anti-WNV therapy is still lagging. To date, there is no proven specific drug or vaccine against WNV in humans and the only recommended treatment is supportive care. Although some small molecules and antibodies capable of neutralizing WNV have demonstrated promising results in clinical studies (phase I and II), additional efficacy trials have not been attempted^16,17^. Thus, continuous efforts are necessary to develop effective anti-WNV therapeutic molecules, such as monoclonal antibodies, nanobodies, peptides, or synthetic compounds. Peptides are becoming promising therapeutics to treat viral infections. Peptide inhibitors can block a specific target (either viral proteins or host receptors) essential for viral attachment, entry, or replication^18^. The possibility of targeting viral attachment protein to contain the viral entry into the cell has emerged as a favorable antiviral strategy^19,20^. We believe that the entry-inhibition could be an effective way to limit WNV infection, specifically by blocking the receptor-binding site (RBS) on its envelope (E) glycoprotein. Moreover, virus can barely develop resistance to entry-inhibiting agents. The resistance to various antiviral agents, mainly to those based on the viral enzyme inhibition strategy, has been reported earlier^21–24^.

Like other members of the *Flaviviridae*, WNV uses its E glycoprotein to mediate the attachment and entry into the susceptible host cell^25–27^. Therefore, the E glycoprotein represents an ideal target for the development of antivirals^28–30^. Protein E is composed of three distinct domains (I to III)^31–33^, of them the immunoglobulin-like domain III (DIII) plays a significant role in mediating viral attachment to the cell receptors^34–41^. Amino acid residues Lys_307_, Thr_330_, and Lys_332_ in DIII were identified as neutralizing epitopes and potential targets for antiviral agents^42,43^, whereas, Glu_306_ and Asp_390_ were proposed essential for glycosaminoglycans binding^44^. The receptor-binding pocket on DIII, responsible for its attachment to human BMECs (hBMECs), was recently identified by us^45^. Changes in the amino acid residues in DIII affect the virulence, which underlies the importance of DIII^37^ and represents a favorable target for the development of entry-inhibiting anti-WNV drug.

Medium size biopharmaceuticals (500 Da–10 kDa) are becoming increasingly popular. Among mid-size biologics, peptides are preferable molecules for being highly selective, efficacious, and relatively safe^46^. They usually have long serum half-life and low production cost^46^. They possess several advantages over small synthetic molecules (< 500 Da) and proteins (> 10 kDa). Small-size synthesized drugs are not necessarily specific to the target, which sometimes leads to serious side effects^47^. The high molecules like antibodies are more target-specific, however, higher production cost, safety, and less membrane permeability are some of the bottlenecks (reviewed in^48^). For the discovery of mid-size peptide-based biomolecules, the combinatorial peptide phage library is being widely used for high-throughput screening, because of its simplicity, commercial availability, and its ability to display peptide sequences with enormous diversity (reviewed in^49,50^). Phage-displayed peptide libraries have been used successfully for epitope mapping^51–53^, drug discovery^54^, protein engineering^55^, and discovery of inhibitory peptides against viral infections^28,56–59^.

In this study, we have screened two combinatorial peptide phage libraries displaying 12-mer linear and structurally constrained 7-mer peptides (with a disulfide bond) to isolate mid-size biomolecules that could inhibit binding of E glycoprotein to the receptors on hBMECs. Peptide libraries were screened against the recently identified RBS (_299_GTTYGVCSK_307_)^45^ of the DIII, which shows strong binding to ~ 15 kDa receptor of the hBMECs. A total of 8 peptides was isolated from two libraries showing binding affinity to DIII. Finally, 4 peptides were able to inhibit the interaction between DIII and endothelial cells, and neutralize the infection in cultured cells. None of these peptides demonstrated toxicity on endothelial cells and hemolytic property. We believe that the peptides (CTKTDVHFC, CIHSSTRAC, CTYENHRTC, and CLAQSHPLC) identified in this study deserve further investigation for their translation into entry-inhibiting mid-sized anti-WNV biomolecules.

## Results

### Selection of DIII binding peptides from combinatorial phage libraries

**Figure 1:**
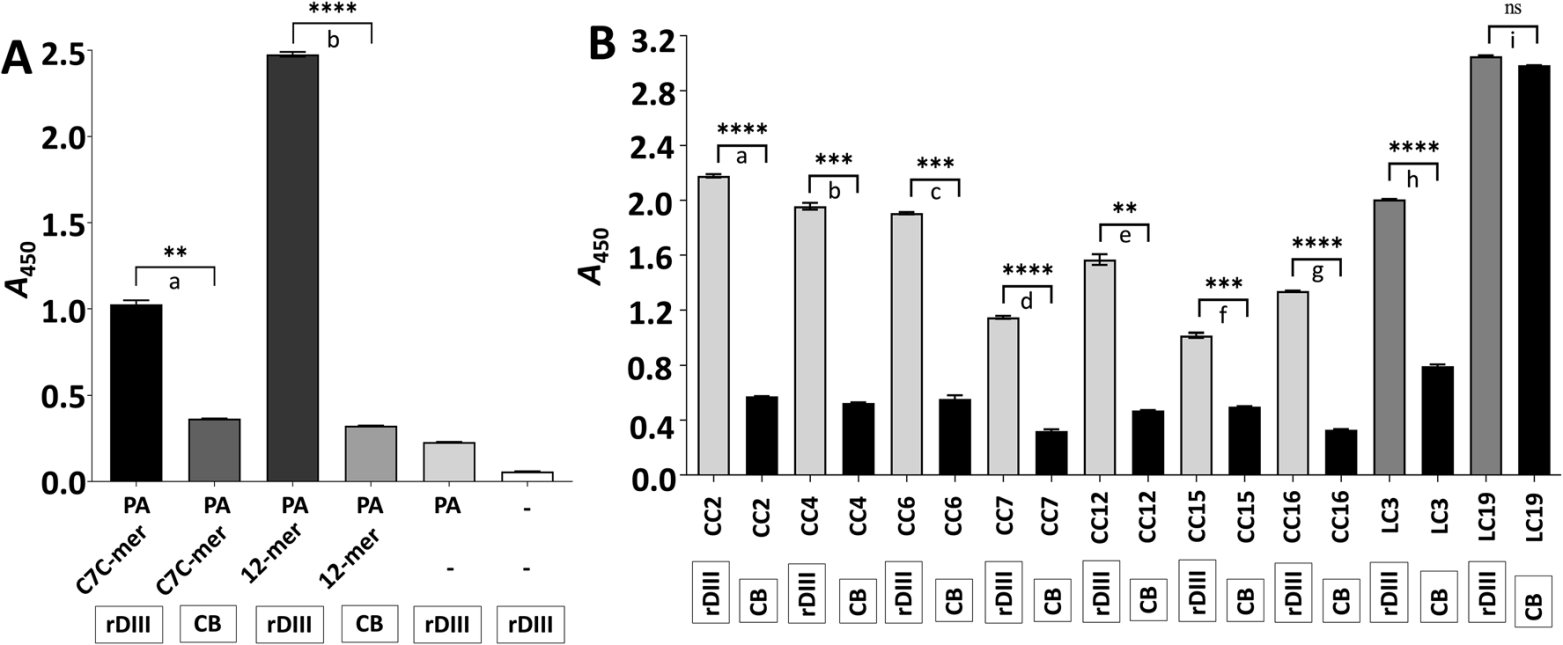
Confirmation of the interaction between rDIII and amplified phages using semi-quantitative ELISA. (**A**) Phage ELISA demonstrating the interaction between rDIII and C7C-mer and 12-mer phages amplified from the last round of biopanning. (**B**) Phage ELISA demonstrating the interaction of individual C7C-mer and 12-mer phage clones to rDIII. Framed reagents were coated into microtiter wells. Data present mean of triplicates with ± S.D. Asterisks indicate statistical significance. A statistical significance difference (P < 0.01, two-tailed P-value) was calculated with paired t-test. Statistics was performed with statistics tool of GraphPad Prism v8.4.3. In Panel (**A**)—a: P = 0.0013; b: P < 0.0001. In Panel (**B**)—a: P < 0.0001; b: P = 0.0002; c: P = 0.0007; d: P < 0.0001; e: P = 0.0014; f: P = 0.0010; g: P < 0.0001; h: P < 0.0001; i: P = 0.037 (ns). *A* – Absorbance; CB – coating buffer; rDIII – recombinant domain III; ns – non-significant; PA – primary antibody, CC – phage clone carrying 7-mer cyclic peptide; LC – phage clone carrying 12-mer linear peptide.

A phage display was employed using two combinatorial Ph.D. libraries (New England Biolabs, USA) to isolate 7-mer cyclic or 12-mer linear peptides, which bind to the DIII of the E protein. Libraries were panned either against RBS (_299_GTTYGVCSK_307_-biotin, ~ 915 Da)^45^ present on the DIII or against recombinant DIII (rDIII; Supplementary Fig. S1). Details of each round of panning are summarized in Table 1. Phages eluted from each round of panning were amplified and purified with PEG/NaCl precipitation. The interaction of the amplified phages from the last round of panning with rDIII was confirmed by semi-quantitative phage ELISA, wherein phages from both libraries showed binding affinity to rDIII (Ph.D.-C7C: *A*_450_ = 1.026; Ph.D.-12: *A*_450_ = 2.477; Fig. 1A). Please note that amplified phages used in ELISA contain a repertoire of all phages eluted in the last panning. Negative controls in ELISA did not show absorbance more than 0.37, which indicates the specific binding of the amplified phages to the rDIII (Fig. 1A).

**Table 1:** Description of each round of panning performed with Ph.D.-C7C and Ph.D.-12 Phage Display Peptide Library.

Round	Solid surface for immobilization of target	Target	Blocking buffer	Amount of phages used to pan (in 100 µL)	Washing buffer	Phage elution
**Ph.D.-C7C Phage Display Peptide Library**						
1st	Pierce Amine-binding, Maleic Anhydride Activated Plates	GTTYGVCSK-biotin (1 µg/well)	0.5% PBST (pH 7.2) + 1 M glycine	2 × 10^11^ PFU in 0.5% PBST (pH 7.2)	0.5% PBST (pH 7.2)	competitive elution with GTTYGVCSK-biotin (9 µg) in PBS (pH 7.2)
2nd	Pierce Amine-binding, Maleic Anhydride Activated Plates	GTTYGVCSK-biotin (1 µg/well)	1% PBST (pH 7.2) + 1 M glycine	2 × 10^11^ PFU in 1% PBST (pH 7.2)	1% PBST (pH 7.2)	competitive elution with GTTYGVCSK-biotin (2 µg) in PBS (pH 7.2)
3rd	CovaLink plates	GTTYGVCSK-biotin (1 µg/well)	CovaBuffer (pH 7.2) + 1 M glycine	2 × 10^11^ PFU in CovaBuffer (pH 7.2) + 1 M glycine	CovaBuffer (pH 7.2) + 1% Tween® 20	(i) non-related peptide in CovaBuffer (pH 7.2) + 1 M glycine; followed by (ii) competitive elution with GTTYGVCSK-biotin (2 µg) in PBS (pH 7.2)
4th	Pierce Nickel Coated Plates	rDIII (0.3 µg/well)	0.1% TBST (pH 7.2) + 1% BSA	2 × 10^11^ PFU in 0.1% TBST (pH 7.2) + 1% BSA	0.1% TBST (pH 7.2) + 20 mM imidazole	0.1% TBST (pH 7.2) + 250 mM imidazole
**Ph.D.-12 Phage Display Peptide Library**						
1st	Pierce Amine-binding, Maleic Anhydride Activated Plates	GTTYGVCSK-biotin (1 µg/well)	0.5% PBST (pH 7.2) + 1 M glycine	2 × 10^11^ PFU in 0.5% PBST (pH 7.2)	0.5% PBST-20 (pH 7.2)	competitive elution with GTTYGVCSK-biotin (9 µg) in PBS (pH 7.2)
2nd	Pierce Amine-binding, Maleic Anhydride Activated Plates	GTTYGVCSK-biotin (1 µg/well)	1% PBST (pH 7.2) + 1 M glycine	2 × 10^11^ PFU in 1% PBST (pH 7.2)	1% PBST (pH 7.2)	competitive elution with GTTYGVCSK-biotin (2 µg) in PBS (pH 7.2)
3rd	CovaLink plates	GTTYGVCSK-biotin (1 µg/well)	CovaBuffer (pH 7.2) + 1 M glycine	2 × 10^11^ PFU in CovaBuffer (pH 7.2) + 1 M glycine	CovaBuffer (pH 7.2) + 1% Tween® 20	(i) non-related peptide in CovaBuffer (pH 7.2) + 1 M glycine; followed by (ii) competitive elution with GTTYGVCSK-biotin (2 µg) in PBS (pH 7.2)
4th	Pierce Nickel Coated Plates	rDIII (0.3 µg/well)	0.1% TBST (pH 7.2) + 1% BSA	2 × 10^11^ PFU in 0.1% TBST (pH 7.2) + 1% BSA	0.1% TBST (pH 7.2) + 20 mM imidazole	0.1% TBST (pH 7.2) + 250 mM imidazole
5th	Pierce Nickel Coated Plates	rDIII (0.3 µg/well)	0.1% TBST (pH 7.2) + 1% BSA	2 × 10^11^ PFU in 0.1% TBST (pH 7.2) + 1% BSA	0.1% TBST (pH 7.2) + 20 mM imidazole	competitive elution with GTTYGVCSK-biotin (2 µg) in PBS (pH 7.2)

CovaBuffer (2 M NaCl, 40 mM MgSO4.7H2O, 0.05% Tween® 20 in PBS).

Amplified phages were then serially diluted up to 10^–10^ and seeded on LB plates supplemented with X-gal and IPTG along with *E. coli* ER2738 (soft agar overlay method) to obtain well-separated phage plaques. Thirty plaques for each library were randomly selected and fragment encoding 7-mer cyclic or 12-mer linear peptides were PCR amplified using vector-specific primers (Supplementary Fig. S2 and Supplementary Table S1) and sequenced. In the case of C7C-mer phage clones, sequences were grouped into 7 clusters (Supplementary Fig. S3A), in which the peptide CTKTDVHFC was the most enriched candidate (68% frequency), followed by CTNANHYFC (12% frequency). The peptides CIHSSTRAC, CMQTQRAHC, CTYENHRTC, CDPRHSKFC, and CLAQSHPLC were presented by 4% frequency. Regarding 12-mer linear phage clones, sequences were clustered into two groups representing SGVYKVAYDWQH (67% frequency) and HYSWSWIAYSPG (33% frequency) peptides (Supplementary Fig. S3B).

Phage display may generate molecules (e.g. peptides, antibodies, etc.) with a high non-specific binding affinity to the solid phase used in panning, substances presented in buffers, or contaminants. Therefore, consecutive panning steps were performed using different solid phases (various plastic surfaces), washing buffers, and different elution strategies to avoid non-specific binders (Table 1). Further, amino acid sequences of peptides were analyzed using the TUPScan tool (SAROTUP server), wherein none of the peptides was classified as a target-unrelated peptide (TUP). Similarly, in a search performed for peptides characterized by other researchers (Peptide search tool in UniProtKB database) no match was found with any of the deposited sequences (Table 2).

**Table 2:** Bioinformatics analysis of identified peptides obtain from phage display.

Phage display peptide library	Phage clone	Amino acid sequence	TUPScan tool	Peptide search tool
Ph.D.-C7C	CC2	CTKTDVHFC	No	No
	CC4	CIHSSTRAC	No	No
	CC6	CMQTQRAHC	No	No
	CC7	CTNANHYFC	No	No
	CC12	CTYENHRTC	No	No
	CC15	CDPRHSKFC	No	No
	CC16	CLAQSHPLC	No	No
Ph.D.-12	LC3	SGVYKVAYDWQH	No	No
	LC19	HYSWSWIAYSPG	No	No

TUPScan tool (http://i.uestc.edu.cn/sarotup3/cgi-bin/TUPScan.pl): a tool of the SAROTUP web server for the prediction of TUPs based on the match with any known TUP motif. Peptide search tool (https://www.uniprot.org/peptidesearch/): a tool for the scan of peptides that match with sequences deposited in the UniProtKB database. CC – phage clone carrying 7-mer cyclic peptide; LC – phage clone carrying 12-mer linear peptide.

Phage clones bearing 7-mer and 12-mer peptides were amplified and purified to test the binding ability of individual clones to the rDIII in semi-quantitative phage ELISA. All representative phage clones bearing 7-mer cyclic peptides showed significantly higher binding affinity to rDIII compared to the negative controls, wherein rDIII was omitted (Fig. 1B). Clones CC2 (phage clone carrying 7-mer cyclic peptide – CC), CC4, and CC6 were the most promising binders (in all cases *A*_450_ > 1.3), while CC15 showed the least affinity to rDIII (*A*_450_ = 0.518). In the case of the clones carrying 12-mer linear peptides, LC3 (phage clone carrying 12-mer linear peptide – LC) showed a significant binding (*A*_450_ = 1.213) to rDIII (Fig. 1B). Although LC19 showed a strong interaction with rDIII (*A*_450_ = 3.051), high absorbance in the negative control (*A*_450_ = 2.985) indicated non-specific binding to the solid-phase (Fig. 1B).

### Production of DIII-blocking peptides in *E. coli* expression system

**Figure 2:**
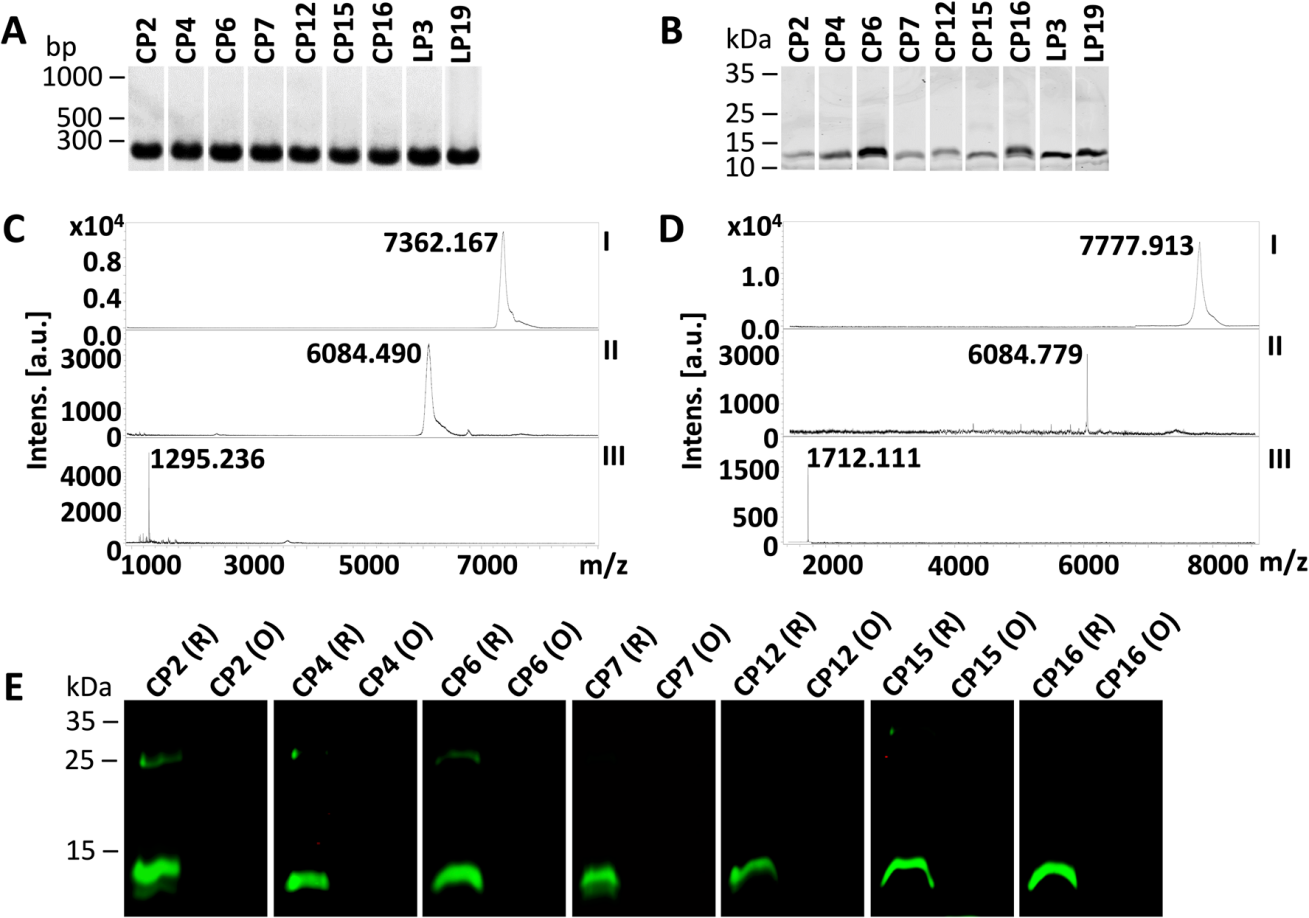
Production of DIII blocking peptides and assessment of the presence of disulfide bond. (**A**) Amplicons encoding tagged peptides resolved on the agarose gel; (**B**) Purified tagged peptides on LDS-PAGE. (**C**) Molecular mass of the representative cyclic peptide (CP2) confirmed by MALDI-TOF MS; (**D**) Molecular mass of the representative linear peptide (LP3) confirmed by MALDI-TOF MS. Predicted masses of tagged peptides (~ 7 kDa) corresponded to observed masses in MALDI-TOF MS (**C** and **D**), however, tagged peptides had slower migration in LDS-PAGE (B). In (**C** and **D**): I – tagged peptide; II – the tag (6xHis tag – 28 aa tag – GGGGS linker) after enterokinase digestion; III – purified peptide after removal of the tag. (**E**) The presence of disulfide bonds in cyclic peptides was confirmed. Any free thiols present in the peptides were blocked with *N*-ethylmaleimid (NEM). Peptides were then either reduced (R) or maintained in oxidized form (O). Both R and O forms were incubated with thiol-reactive IRDye 800CW Maleimide and then separated on non-reducing LDS-PAGE. In the peptide, if thiols are occupied with the disulfide bond, they remain unblocked and get reduced with TCEP. Free thiols in reduced peptides are then labeled giving a green signal. In the oxidized form, no free thiols are available, thus no labeling occurs (no green signal).

The 7-mer and 12-mer peptides from the phage clones used in phage ELISA, mentioned above, were produced in the *E. coli* expression system. All peptides were N-terminal tagged, and the sequence of the overexpression cassette was as follows: 6xHis tag – *Bam*HI restriction site (GS) –28 aa tag (devoid of cysteine and methionine residues) – Factor Xa cleavage site (IEGR) – *Sal*I restriction site (VD) – GGGGS linker – enterokinase cleavage site (DDDDK) – [alanine (A)] – 7-mer or 12-mer peptide sequence (7-mer sequences were flanked with cysteine at both ends) – GGGS linker – stop codon (Supplementary Fig. S4). Please note that, all peptides had the C-terminal GGGS linker to mimic their presentation on the phage pIII protein. DNA encoding peptide sequence was ligated into the expression vector (Supplementary Fig. S5) and electroporated to *E. coli* Shuffle. Correct insertion of DNA in the transformants confirmed with PCR and the purity of all tagged peptides confirmed with LDS-PAGE are presented in Fig. 2A and B. Molecular masses of the representative cyclic and linear peptides assessed by MALDI-TOF MS are presented in Fig. 2C and D. The molecular masses of all tagged peptides judged with MALDI-TOF MS are presented in Supplementary Fig. S6A. Peptides were cleaved off the N-terminal tag using enterokinase and the tag was removed with nickel affinity chromatography. Cleaved ACX_7_CGGGS or X_12_GGGS peptides were used for the assessment of rDIII binding ability and rDIII blocking potential. Precise molecular masses of peptides (without the tag) judged with MALDI-TOF MS are presented in Supplementary Fig. S6B. The observed molecular masses of all peptides were matched with theoretical masses predicted by Geneious Pro v9.1.8 software.

### ACX_7_CGGGS peptides are in cyclic form

The peptides of Ph.D.-C7C phage library are presented on the phage pIII protein in cyclic form due to the formation of the disulfide bond between two cysteine residues. Thus, cyclization is essential to maintain the functionality of the peptides. To achieve the formation of the disulfide bonds, protein constructs were overexpressed in *E. coli* Shuffle, which are having enhanced capacity to correctly fold proteins with disulfide bonds in the oxidized cytoplasm. The presence of disulfide bond was verified with thiol-reactive maleimide labeling. In brief, free thiols in the protein constructs were blocked, the disulfide bond in the protein was cleaved with a reducing agent, and the revealed thiol groups were labeled with IRDye Maleimide. Non-reduced (oxidized) protein samples were used as a control. The reduced form of the protein was readily conjugated with maleimide IRDye, while the oxidized form of the same protein failed to get labeled (Fig. 2E). These results confirm the presence of the disulfide bonds in ACX_7_CGGGS peptides, which indicates that peptides are in cyclic form.

### Screening of the binding ability of peptides to rDIII

**Figure 3:**
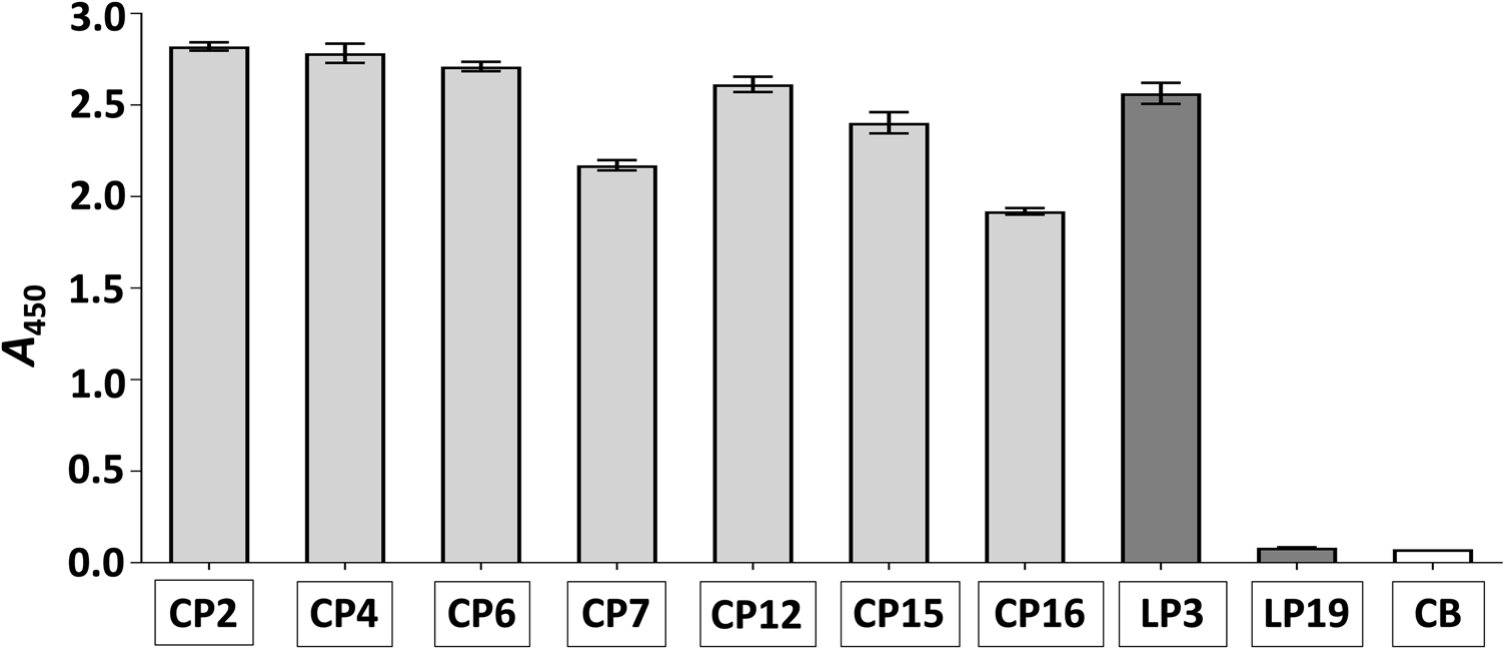
Assessment of the interaction between peptides and rDIII. Framed reagents were coated into microtiter wells (Nunc CovaLink NH plates), incubated with rDIII, and the interaction was detected with HisProbe-HRP conjugate. Data present mean of triplicates with ± S.D after subtraction of negative control (rDIII was excluded from the assay). CB – coating buffer (1-ethyl-3-(3-dimethylamino-propyl)-carbodiimide, EDC plus sulfo-N-hydroxysuccinimide, sulfo-NHS); CP – cyclic peptide; LP – linear peptide. Please note that, all wells were blocked with a blocking buffer (0.5% BSA in PBS) after overnight coating.

This screening was necessary to show that 7-mer cyclic peptides (CP) and 12-mer linear peptides (LP) produced by us possess DIII binding potential, which is crucial for blocking the interaction between DIII and hBMECs. The screening was also important to show that the binding ability of peptides is retained in the absence of pIII phage protein. The screening was performed with ELISA, wherein the peptides were coated into microtiter wells through their C-terminus. For all 7-mer cyclic peptides, the absorbance > 1.9 acknowledge that these peptides represent strong DIII binders (Fig. 3). From two 12-mer linear peptides, only LP3 showed a strong binding to rDIII (*A*_450_ = 2.564), whereas LP19 did not retain its binding ability to rDIII (*A*_450_ = 0.082, Fig. 3). Therefore, LP19 was excluded from further assays.

### Peptides are able to block the binding of rDIII to hBMECs (ELISA)

**Figure 4:**
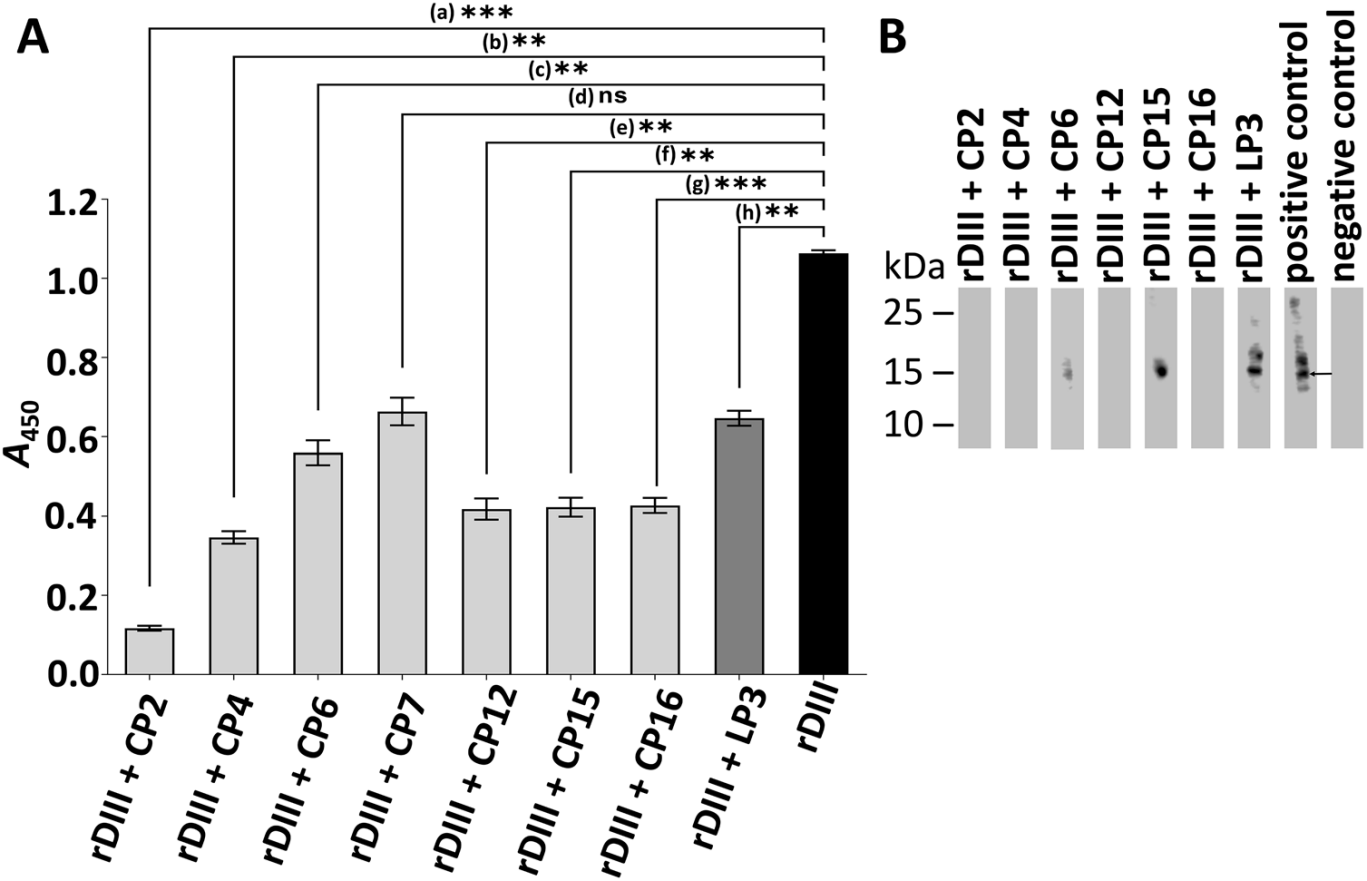
Assessment of the blocking ability of peptides on proteins of hBMECs. (**A**) Blocking of the interaction between proteins of hBMECs and rDIII by ELISA. Proteins of hBMECs were coated into microtiter wells. Data present mean of triplicates with ± S.D after subtraction of negative control (rDIII excluded from the assay). A statistically significant difference (P < 0.01, two-tailed P-value, GraphPad Prism v8.4.3.) was calculated by paired t-test compared to the positive control (interaction between rDIII and hBMECs proteins). a: P = 0.0002; b: P = 0.0010; c: P = 0.0058; d: P = 0.0101 (ns); e: P = 0.0015; f: P = 0.0017; g: P = 0.0004; h: P = 0.0021. *A* – Absorbance. (**B**) Blocking of the interaction between proteins of hBMECs and rDIII by Western blot. Arrow shows interaction between rDIII and ~ 15 kDa receptor of hBMECs. Positive control – unblocked rDIII incubated hBMECs proteins. Negative control – rDIII was omitted from the protocol. rDIII – recombinant DIII; CP – 7-mer cyclic peptide; LP – 12-mer linear peptide.

The 7-mer cyclic and 12-mer linear peptides that showed strong binding ability to rDIII (Fig. 3) were assessed for their potential to inhibit the interaction between rDIII and proteins of hBMECs. Before the blocking of rDIII-hBMECs interaction, the minimum concentration at which rDIII is able to interact with hBMECs proteins was determined. Absorbance higher than 1.0 was set as a threshold to select the minimal concentration of rDIII. It was observed that a minimum of 2 µg (0.15 nM) of rDIII was sufficient to detect the interaction (Supplementary Fig. S7). Thus, rDIII at this concentration (0.15 nM) was pre-incubated with a tenfold molar excess (1.5 nM) of each peptide prior to incubation with hBMECs proteins in ELISA. Among cyclic peptides, CP2 showed the highest inhibition (*A*_450_ = 0.117, 89% inhibition) followed by CP4 (*A*_450_ = 0.346, 67% inhibition), CP12 (*A*_450_ = 0.418, 61% inhibition), CP15 (*A*_450_ = 0.423, 60% inhibition), CP16 (*A*_450_ = 0.427, 60% inhibition), and CP6 (*A*_450_ = 0.56, 47% inhibition; Fig. 4A). Absorbance in the positive control (without pre-blocking of rDIII) was *A*_450_ = 1.064. CP7 showed 37% inhibition, however, the blocking of the interaction was not significantly different (P = 0.0101) from the positive control. Therefore, CP7 was not included in further assays. When rDIII was pre-incubated with a linear peptide LP3, 39% inhibition (*A*_450_ = 0.647) in its interaction with hBMECs was observed (Fig. 4A).

### Peptides are able to block the binding of rDIII to hBMECs (Western blot)

Western blot was also employed to screen ability of peptides to inhibit the interaction between rDIII and low molecular weight receptors (~ 15 kDa) of hBMECs. Considering that rDIII has shown strong binding affinity with ~ 15 kDa receptor in our recent study^45^, we suppose that inhibition of this interaction would block binding of the E protein on the endothelial cells. hBMECs receptors transblotted on the nitrocellulose (NC) membrane were excised as shown in Supplementary Fig. S8 and used in Western blot. Before the blocking of rDIII interaction with ~ 15 kDa hBMECs receptor, the minimum concentration of rDIII required to detect visible interaction to the endothelial receptor was assessed. It was shown that 2.5 µg (0.19 nM) of rDIII were necessary to detect the visible interaction (Supplementary Fig. S9). Thus, in the blocking assay, 2.5 µg (0.19 nM) of rDIII were pre-incubated with a tenfold molar excess (1.9 nM) of each peptide prior to incubation with hBMECs strips. The inhibition of the interaction was confirmed in the case of CP2, CP4, CP12, and C16 (Fig. 4B). CP6, CP15 and LP3 did not show clear inhibition of the interaction (Fig. 4B). Therefore, these peptides were excluded from the further assays. Positive control (unblocked rDIII) included in the Western blot showed a signal at ~ 15 kDa, while in the negative control (rDIII excluded from assay), no signal was observed (Fig. 4B).

### Peptides are able to block the binding of rDIII to hBMECs (immunocytochemistry)

**Figure 5:**
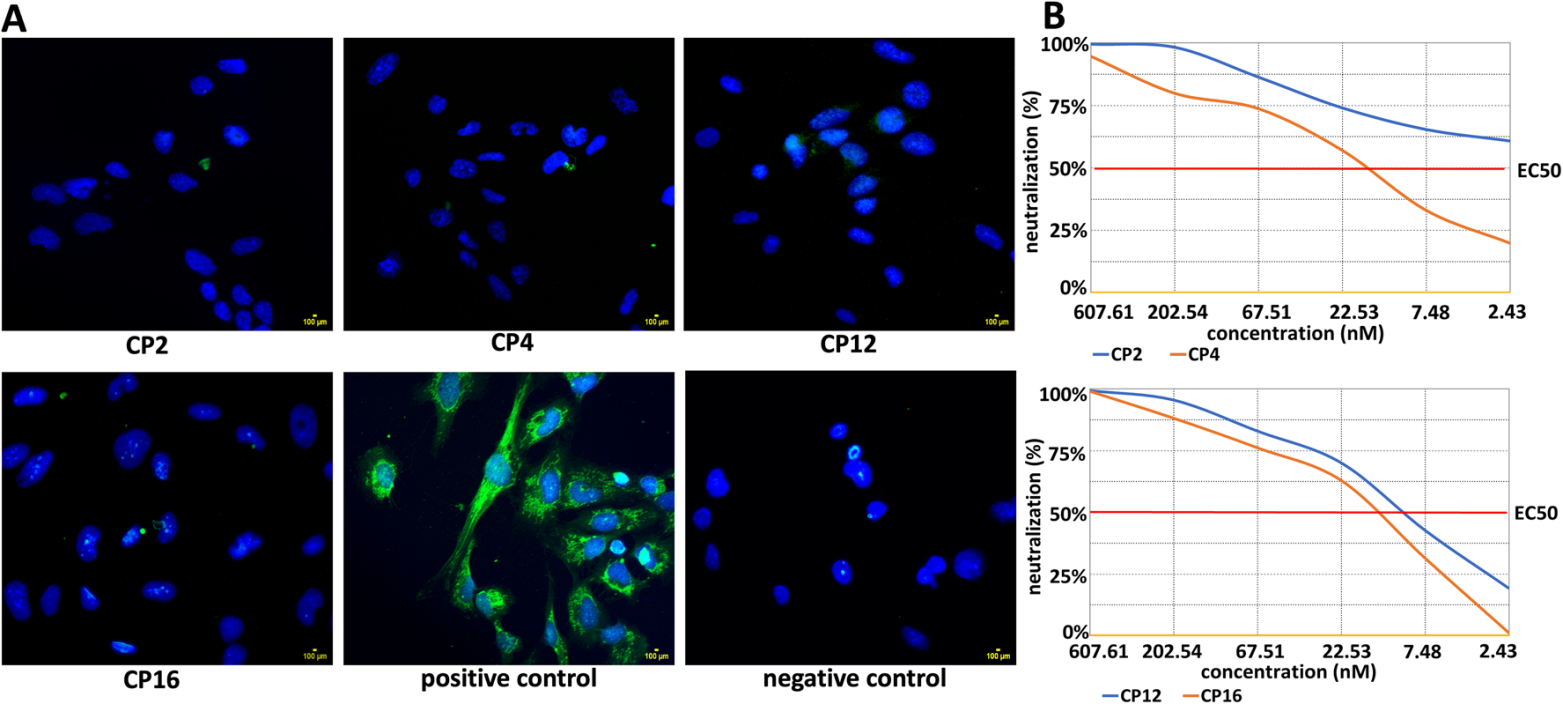
Blocking of the adhesion of rDIII on cultured endothelial cells and neutralization assay. (**A**) Blocking of the adhesion of rDIII on endothelial cells. Nuclei are stained with DAPI. The assay was performed in triplicates. Positive control – rDIII was incubated with the cells. Negative control – rDIII was excluded from the assay. CP – 7-mer cyclic peptide. (**B**) Assessment of the ability of peptide to block infection in cultured cells (neutralization assay). Neutralization of the infection of virus like particle carrying *Fluc* gene by CP2, CP4, CP12 and CP16. The amount of VLP entering the target cells was calculated by detecting the expression of luciferase, which then used to measure the neutralizing ability of the peptides, expressed in half maximal effective concentration (EC_50_). Neutralizing capacity of each CP was observed as follows: CP2 – EC_50_ > 7290 (17.3 ng/ml, 2.43 nM), CP4 – EC_50_ 1144 (119.4 ng/ml, 17 nM), CP12 – EC_50_ 1811 (73.3 ng/ml, 10.4 nM) and CP16 – EC_50_ 1270 (104.7 ng/ml, 14.8 nM).

Based on ELISA and Western Blot results, CP2, CP4, CP12, and CP16 were selected for further assessment by immunocytochemistry. To block the adhesion of rDIII to the culture of hBMECs, the minimum concentration of rDIII needed to detect visible interaction with the endothelial cells was determined. Five micrograms (0.39 nM) of rDIII were sufficient to visualize adhesion of rDIII on endothelial cells culture in immunocytochemistry (data not shown). rDIII was pre-blocked with a tenfold molar excess (3.9 nM) of each peptide before incubation with the cells. All four peptides were able to inhibit the adhesion of rDIII on the endothelial cells, which ensures the blocking potential of selected anti-WNV peptides (Fig. 5A).

### Peptides are able to block infection in cultured cells

The ability to block the infection was assessed by the neutralization test (Fig. 5B, Supplementary datasets 1 and 2). Among four peptides, CP2 showed the best neutralization capacity with EC_50_ > 7290 (17.3 ng/ml) corresponding to 2.43 nM. The CP12 showed 50% neutralization of the infection at 10.4 nM (73.3 ng/ml, EC_50_ 1811). Remaining two peptides, CP16 and CP4, were able to neutralize the VLP at 14.8 nM (104.7 ng/ml, EC_50_ 1270) and 17 nM (119.4 ng/ml, EC_50_ 1144), respectively (Fig. 5B, Supplementary dataset 2).

### Cytotoxic and hemolytic activity of peptides

**Figure 6:**
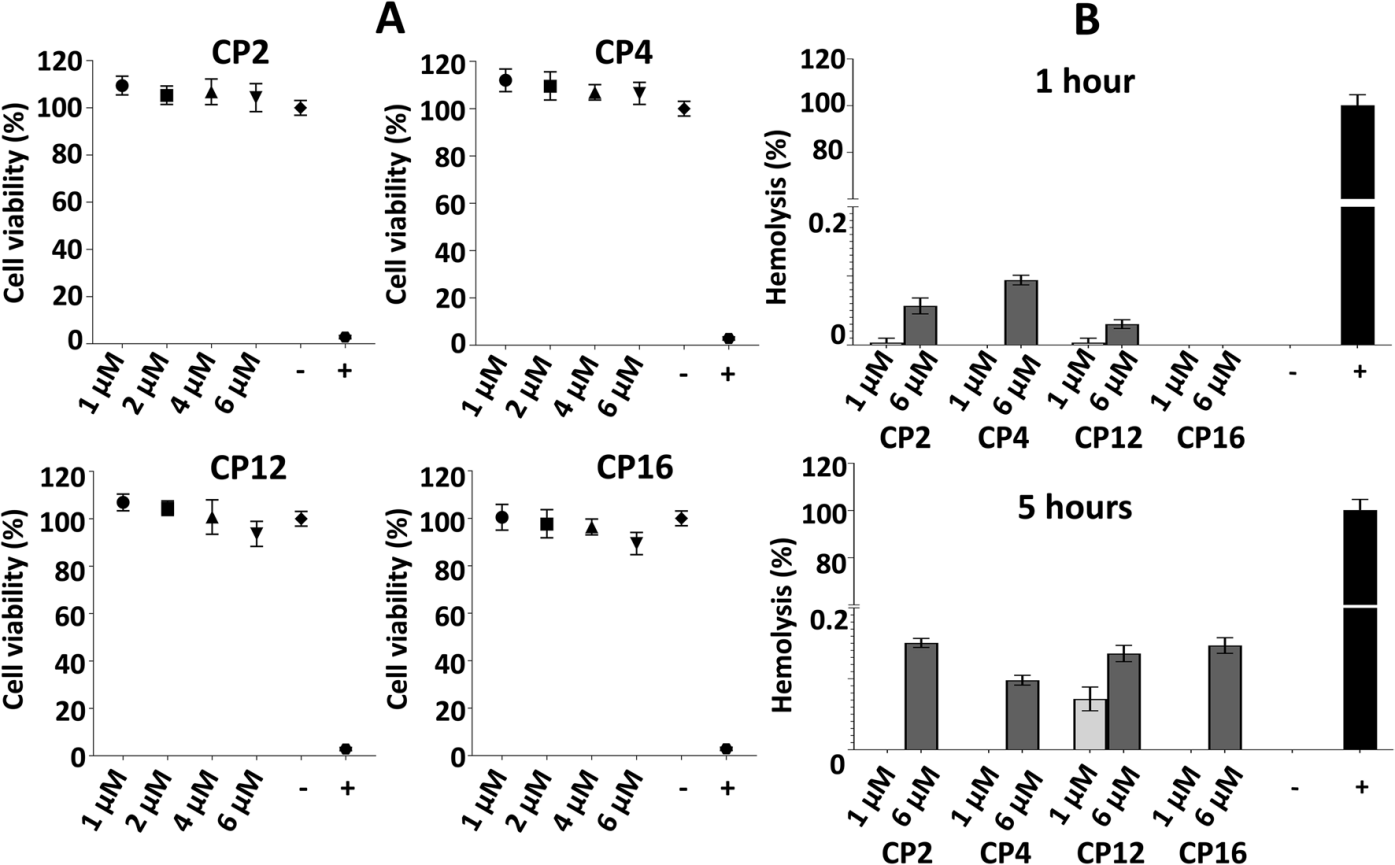
Cytotoxic and hemolytic activity of peptides. (**A**) Cytotoxicity of DIII blocking peptides assessed by XTT test after 24 h of incubation at different concentrations. A statistically significant difference (P < 0.01, two-tailed P-value) was calculated by unpaired t-test with Welch´s correction compared to the negative control (untreated cells). None of the peptides showed significant cytotoxicity. Statistics was performed with GraphPad Prism v8.4.3. “-” – negative control (untreated hBMECs); “+” – positive control (cells treated with 0.1% Triton X-100); CP – 7-mer cyclic peptide. (**B**) Assessment of hemolytic activity of peptides. Suspension of sheep erythrocytes was incubated with peptides at two different concentrations. No hemolytic effect (no release of hemoglobin) of peptides was observed. “-” – negative control (untreated sheep erythrocytes); “+” – positive control (sheep erythrocytes treated with 0.1% Triton X-100); CP – 7-mer cyclic peptide.

Four DIII blocking peptides (CP2, CP4, CP12, and CP16) were tested by XTT for cytotoxic effect induced in eukaryotic cells. First, the cell viability was standardized in untreated (100% viability) and Triton treated cells (< 2.8% viability; Fig. 6A). The toxicity of each peptide was determined at different concentrations (1 μM, 2 μM, 4 μM, and 6 μM). None of the peptides was toxic for eukaryotic cells even at 6 μM concentration (Fig. 6A). Among all peptides, the maximum decrease in viability was observed in the case of CP16 (6 μM, 10.6% decrease), however, the reduction was not significant to designate CP16 as a toxic peptide (P = 0.1158).

Further assessment of toxicity was performed by evaluating the hemolytic activity of peptides on sheep red blood cells (RBCs). Hemolysis > 10% was set as a threshold to designate the peptide with hemolytic activity. Hemolysis was measured at two different concentrations (1 μM and 6 μM) at two different time points (1 h and 5 h). None of the DIII WNV blocking peptides (CP2, CP4, CP12, and CP16) showed hemolytic activity as the maximum release of the hemoglobin was < 0.15% (Fig. 6B).

## Discussion

The neuroinfections caused by viruses represent a severe threat worldwide. Till to date, antiviral therapeutics are available only against few viruses (reviewed in^60–63^). In the case of WNV infection, no specific antiviral therapies are available and the treatment is restricted to supportive care.

Different stages in the viral life cycle can be targeted to develop antiviral molecules, however, inhibition of the initial steps, the binding and entry into the host cells, is an attractive strategy^64,65^. In case of flaviviruses, E glycoprotein is the only protein that mediates the binding of viral particles to the host cells. It is composed of 3 ectodomains (DI – DIII)^26,32,66^, while the DIII is a receptor-binding functional determinant for the viral entry^31,36,42,43,66–68^. This makes DIII a good candidate to stop the viral entry and a favorable target for the development of anti-flavivirus therapy. The binding of DIII to endothelial cells of the brain vasculature was also confirmed in our previous study^45^. hBMECs were selected as target cells in this study since they represent a primary barrier to viral dissemination into the CNS. It was demonstrated that hBMECs are sensitive to WNV and can facilitate entry of the cell-free virus into the CNS without compromising the BBB integrity, suggesting a key role of the endothelial layer in the virus spread via the hematogenous route^9,69^. Therefore, it is supposed that the adhesion of WNV to hBMECs is one of the crucial steps in flavivirus neuroinvasion.

We believe that the entry-inhibiting strategy could be an effective way to limit WNV neuroinvasion. The viral entry-inhibition strategy is adopted against various viruses (reviewed in^70–72^). One possible advantage of this entry-inhibiting approach is that it becomes difficult for the virus to develop resistance to entry-inhibiting agents. The resistance to various antiviral agents, mainly to those based on the viral-enzyme inhibition strategy, has been reported earlier^21–24^. Compared with inhibitors targeting viral enzymes, entry inhibitors can directly bind to virions in the blood without the need for penetration into cells. Furthermore, since the entry-inhibiting compounds block the very first step of the viral life cycle wherein the host cell membrane permeability is not necessary, they have the potential to be less toxic. Several anti-WNV monoclonal antibodies were developed previously as entry-blocking agents^73–76^, however, their cross-reactivity was the major limiting factor. Therefore, more specific and efficacious therapeutic tools against WNV are highly required. The high specificity can be achieved by selecting the molecules specifically against RBSs. Thus, the RBS _299_GTTYGVCSK_307_ located at the N-terminus of DIII^45^ was targeted to develop plausible entry-blocking peptides in this study by panning two different combinatorial phage display libraries. Although the antiviral peptides targeting E protein of various flaviviruses were isolated previously^28,59,77,78^, anti-WNV peptides^28^ were generated by using full-length E protein. E glycoprotein is a large molecule (53–60 kDa)^79^ with several surface-exposed regions not taking part in the receptor-binding. Panning of combinatorial libraries against this target may lead to the selection of peptides that can strongly bind to the E protein, however, fail to occupy RBSs.

Antiviral peptides can be obtained employing various approaches such as natural sources (e.g. from plants), computational methods (e.g. molecular docking), or biological source (e.g. display techniques). From all available display techniques, phage display represents a widely used methodology for the selection of peptides with selective affinity for a specific target. The success of a selection (a panning) in display technologies relies on the complexity of the library. The greater is the diversity of clones, the more likely the library contains sequences that will be a target-specific binder. Combinatorial phage display libraries used in this study consist of 10^9^–10^10^ random variants of peptide sequences displayed on pIII phage protein in 5 copies represent valuable tools for the selection of peptides. Both combinatorial phage libraries (C7C-mer and 12-mer) are constructed using the same M13 bacteriophage backbone, however, the 12-mer library can be propagated more efficiently than the C7C-mer library^80^. On the other hand, studies have reported that high-affinity ligands are readily selected from the loop-constrained cyclic peptide libraries, like C7C-mer, than from linear libraries^81–83^. Thus, both combinatorial libraries were used in this study to obtain effective DIII blocking peptides.

In the present study, among 9 unique panned peptides (7 cyclic and 2 linear), 8 peptides showed specific binding to rDIII (Fig. 3). Among those 8 peptides only four cyclic peptides (CP2 – CTKTDVHFC, CP4 – CIHSSTRAC, CP12 – CTYENHRTC, CP16 – CLAQSHPLC) were able to block the interaction between rDIII and hBMECs judged by ELISA, Western blot and immunocytochemistry (Fig. 4 and Fig. 5A). Those four peptides also inhibited the infection in cultured cells assessed by neutralization assay (Fig. 5B). None of the linear peptides showed potential to inhibit the interaction. The fact that other phage display-selected peptides (CP6 – CMQTQRAHC, CP7 – CTNANHYFC, CP15 – CDPRHSKFC, LP3 – SGVYKVAYDWQH) with good DIII binding activity (in all *A*_450_ > 1.5) did not block the interaction between rDIII to hBMECs was not surprising (Fig. 4). It may be possible that those peptides did not occupy the RBSs sufficiently to be able to hinder rDIII-receptor interaction.

From overall experimental approach in the present study, 4 cyclic peptides and none of the linear counterpart were able to block the interaction between DIII and hBMECs. Historically, structurally constrained peptide-based drugs are more successful as therapeutics than linear peptides^84–86^, and it is tempting to speculate that our finding is in line with this fact. Cyclic peptides have better binding affinity toward the target molecules because of the conformation^87^, which allow them to occupy the larger surface area of RBSs necessary to hinder the ligand-receptor interaction effectively^88,89^. Moreover, cyclic peptides are resistant to proteolysis, demonstrate effective crossing of the membranes, and are less toxic^90,91^. To rule out the possible toxicity of the 4 cyclic peptides generated in the present study, the XTT and hemolytic test were used. The therapeutic antiviral peptide should be specific for virus-related processes with no or fewer effects on cell metabolism and should not demonstrate acute or permanent toxicity against the host. It is generally accepted that dehydrogenase activity more than 70% compared to untreated control (ISO 10993–5: Biological evaluation of medical devices—Part 5: Tests for in vitro cytotoxicity) and hemolysis < 10%^92^ define peptide as a safer candidate for translation of the biomolecules to therapy. In our study, all 4 cyclic peptides showed no signs of toxicity to hBMECs (Fig. 6A), and hemolytic activity (Fig. 6B) even at the concentration of 6 µM. This concentration is approximately 1500 times higher than the concentration required for blocking of rDIII-hBMECs interaction evaluated by immunocytochemistry, and approximately 2469 times higher than the concentration of CP2 required to neutralize VLP (EC_50_ > 7290, 2.43 nM) in neutralization assay. In case of other three CPs, the concentrations required to exhibit EC_50_ were ranged between 10–17 nM only. Overall results indicate that CPs can neutralize the infection in vitro at nanomolar concentration with no toxic and hemolytic effect even at several 100-fold higher concentration.

In conclusion, the blocking peptides identified in this study (CTKTDVHFC, CIHSSTRAC, CTYENHRTC, CLAQSHPLC) could present the basic building block for the development of new entry-inhibiting antivirals against WNV neuroinvasion since specific therapy is still not available. The experimental approach presented here may, potentially, serve as a template to design mid-size antiviral therapeutics against other emerging viruses.

## Methods

### Human brain microvascular endothelial cells culture

hBMECs were obtained from Merck/Millipore (Czech Republic). Details of hBMECs cultivation are presented in Supplementary Method S1.

### Protein extraction of hBMECs

The confluent monolayer of hBMECs was washed twice with PBS (pH 7.4) and scraped. Proteins were extracted under native conditions as presented in Supplementary Method S2. Proteins were stored at −80 °C until use.

### Synthesis of recombinant domain III

rDIII of the E protein of WNV was overexpressed in the *E. coli* M15 expression system (Qiagen, Germany) as described in our previous publication^45^. Highly purified protein was stored at −20 °C in 35% glycerol until use. The concentration of rDIII was measured by the Bradford method.

### Selection of phage-displayed peptides with affinity to the receptor-binding site of DIII

Phage display was performed using Ph.D.-C7C and Ph.D.-12 Phage Display Peptide libraries according to the manufacturer´s instructions (New England Biolabs). Libraries were panned either against ~ 915 Da synthetic analog of RBS (_299_GTTYGVCSK_307_-biotin) present on DIII or against rDIII. Several rounds of surface panning were performed using different types of plates and various strategies of phage elution. Details of each panning are presented in Table 1. In general, synthetic peptide (RBS) or rDIII were coated in 96-well plates overnight at 4 °C (as per manufacturer’s instructions) and non-specific binding sites were blocked with blocking buffer for 1 h at room temperature. After three washings with washing buffer, 2 × 10^11^ phages were allowed to interact with immobilized target for 1 h at room temperature. The wells were then washed 15 times with the same washing buffer and phages were eluted as presented in Table 1. After each elution, number of phages present in eluate were calculated with the plate titration method and the rest of the eluate was subjected for amplification of phages in *E. coli* ER2738 (New England Biolabs) for 4.5 h at 37 °C. Amplified phages were precipitated from cleared *E. coli* culture with PEG/NaCl precipitation as per the manufacturer’s instructions (New England Biolabs). After the last round of panning, phages in eluate were amplified, precipitated, and their binding ability to rDIII was judged by phage ELISA (details are described in Supplementary Method S3).

Amplified phages were then plated on LB agar plates containing 1 mM IPTG (Fermentas, Slovakia) and 1 mM X-gal (Sigma, USA) and 30 well-separated phage plaques were randomly picked and propagated as per the manufacturer’s instructions (New England Biolabs). Individual phage clones were subjected to DNA sequencing and phage ELISA.

### Isolation of phage DNA, sequencing, and in silico peptide sequences analysis

Genomic DNA from each phage clone was isolated as per the manufacturer’s instructions (New England Biolabs). Sequence encoding phage-displayed peptide was amplified with PCR (vector-specific primers are presented in Supplementary Table S1) and sequenced on ABI PRISM 3100-Advanced Genetic Analyzer (Thermo Fisher Scientific, Slovakia). The amino acid sequences of peptides (presented on phage clones) were deduced with the help of Geneious Pro v9.1.8 software (Biomatters, USA). The bioinformatics analysis of identified peptide sequences was performed to exclude any non-specific binder (TUPs) with the help of the SAROTUP server (http://i.uestc.edu.cn/sarotup3/index.html)^93^. The UniProtKB database (Peptide search tool, https://www.uniprot.org/peptidesearch/) was used to search possible sequence match in the public repository.

### Assessment of binding ability of individual phage clone with rDIII

Amino acid sequences of the peptides (presented on pIII phage protein) deduced above were aligned and grouped as per sequence homology. A representative phage clone from each group was amplified in *E. coli* ER2738 and purified with PEG/NaCl precipitation as described above. Each phage clone was then analyzed for its binding ability to rDIII using phage ELISA (Supplementary Method S4).

### Production of 7-mer cyclic and 12-mer linear peptides

The 7-mer cyclic and 12-mer linear peptides from the selected phage clones were produced in the *E. coli* SHuffle expression system (New England Biolabs). All peptides were flanked with GGGS sequence at C-terminus (e.g. X_12_GGGS), while 7-mer cyclic peptides contain additional alanine residue at N-terminus (ACX_7_CGGGS) as suggested by the manufacturer of Ph.D. libraries (Supplementary Figure S4). Nucleotide sequences of amplified fragments used for the digestion and ligation into the pQE-30-UA-mCherry-STOP expression vector (Supplementary Figure S5) are summarized in Supplementary Table S2. The protein constructs were overexpressed in the *E. coli* SHuffle T5 strain. Detail steps of cloning, transformation of *E. coli*, selection of clones, protein overexpression, purification, and quality control are presented in Supplementary Method S5, S6, S7, and S8. The concentration of recombinant protein constructs was measured with NanoDrop One (Thermo Fisher Scientific). Aliquots of purified protein constructs were vacuum-dried and stored at room temperature until further use.

### Removal of the tag and recovery of peptides

The N-terminal tag (6xHis tag – 28 aa tag – GGGGS linker) was cleaved with His tagged bovine enterokinase as per the manufacturer´s instructions (GenScript Biotech, USA). Please note that tagged peptides were designed with the enterokinase cleavage site (highly specific DDDDK sequence). In short, 50 µg of tagged peptides were incubated with 5 µL of 10X cleavage/capture enterokinase buffer (200 mM Tris–HCl, 500 mM NaCl, 20 mM CaCl2; pH 7.4) and 2 µL of enterokinase (diluted 1:5 [v/v] in dilution/storage enterokinase buffer; 20 mM Tris–HCl, 200 mM NaCl, 2 mM CaCl2, 50% glycerol; pH 7.4) at 22 °C for 16 h. The mixture was incubated with His Mag Sepharose Ni beads (Thermo Fisher Scientific) for 2 h at room temperature with gentle agitation. During the incubation, the tag (6xHis tag – 28 aa tag – GGGGS linker) and the enterokinase (please note that the enzyme is his tagged) were bound to the beads, while the unbound part containing ACX_7_CGGGS or X_12_GGGS peptide remained in solution. The solution containing peptides was collected in a new tube. The purity of the peptide was checked by MALDI-TOF MS (as described in Supplementary Method S8).

### Assessment of the disulfide bonds in tagged peptides

The presence of disulfide bonds in tagged 7-mer cyclic peptides was judged with thiol-reactive IRDye 800CW Maleimide (LI-COR Biosciences, USA) labeling after the reduction of tagged peptides with tris(2-carboxyethyl)phosphine (TCEP, Sigma). Detail steps are presented in the Supplementary Method S9. After labeling reaction, tagged peptides were separated with LDS-PAGE with minor modifications (Supplementary Method S10) and signals were captured on the Odyssey CLx imaging system (LI-COR Biosciences).

### Assessment of the binding of peptides to rDIII

The 7-mer cyclic and 12-mer linear peptides were covalently bound in CovaLink NH microwell plates and their binding to rDIII was corroborated by ELISA. Details are presented in Supplementary Method S11.

### Blocking of the interaction between rDIII and proteins of hBMECs

First, concentration-dependent ELISA and Western blot were performed to get the minimum concentration of rDIII required to show its interaction with hBMECs proteins. Details are presented in Supplementary Method S12 and Supplementary Method S13. Subsequently, the ability of 7-mer and 12-mer peptides to block the interaction between rDIII and proteins of hBMECs was tested. A tenfold molar excess of peptides was incubated with rDIII for 1 h at room temperature. Such pre-incubated rDIII was subsequently used in the assays. Details of both methods are described in Supplementary Method S12 and Supplementary Method S13.

### Blocking of the adhesion of rDIII on cultured hBMECs

Cells were cultured as described in Supplementary Method S1. First, concentration-dependent immunocytochemistry was performed to reveal the minimum concentration of rDIII required to detect its adhesion to the endothelial cells. Details are presented in Supplementary Method S14.

After that, the ability of peptides to block the adhesion of rDIII to the culture of hBMECs was judged. A tenfold molar excess of each peptide was incubated with rDIII for 1 h at room temperature. Pre-incubated rDIII was subsequently used in the assay. Details are described in the Supplementary Method S14.

### Peptide cytotoxicity assay

The toxicity of peptides was determined by the XTT test (AppliChem, USA) as per the manufacturer’s instructions. Details are presented in Supplementary Method S15.

### Assessment of the hemolytic activity

Hemolysis assay was performed according to the method of Bender et al.^94^. Details are presented in Supplementary Method S16.

### Assessment of the ability of peptide to block infection in cultured cells

The ability to block infection in cultured cells was assessed using virus like particles (VLP) that possess C, M, and E proteins, and carry luciferase reporter gene. Details of the neutralization test are presented in Supplementary Method S17. In short, the peptides were serially diluted, which then mixed with VLP (400–500 TCID_50_/ml) and incubated for 90 min. Cultured cells were infected with the peptide-VLP mixture and incubated for 48 h. The amount of VLP entering the target cells was calculated by detecting the expression of luciferase, which then used to measure the neutralizing ability of the peptides, expressed in half maximal effective concentration (EC_50_).

## Supplementary Information

Supplementary Information 1.

Supplementary Information 2.

Supplementary Information 3.

## Data Availability

Data generated in this study are available from the corresponding author on reasonable request.
