# Supplementary material for: Development of peptides targeting receptor binding site of the envelope glycoprotein to contain the West Nile virus infection
Source: Sci Rep. 2021 Oct 11;11:20131. doi: 10.1038/s41598-021-99696-w (PMC8505397; doi:10.1038/s41598-021-99696-w)
Supplement: Supplementary file 3 — Supplementary Information 3. [file 41598_2021_99696_MOESM3_ESM.pdf]

Development of peptides targeting receptor binding site of the envelope glycoprotein to contain the West Nile virus infection

Patrícia Mertinková[1], Evelína Mochnáčová[1], Katarína Bhide[1], Amod Kulkarni[1,2], Zuzana Tkáčová[1], Jana Hruškovicová[1], Mangesh Bhide[1,2*]

[1]Laboratory of Biomedical Microbiology and Immunology, The University of Veterinary Medicine and Pharmacy in Košice, Komenského 73, 04181, Košice, Slovakia.

[2]Institute of Neuroimmunology of Slovak Academy of Sciences, Dubravska cesta 9, 84510, Bratislava, Slovakia.

***Corresponding author:** Assoc. Prof. Mangesh Bhide, PhD., Laboratory of Biomedical Microbiology and Immunology, The University of Veterinary Medicine and Pharmacy in Košice, Komenského 73, 04181, Košice, Slovakia

e-mail: bhidemangesh@gmail.com

Phone - +421 918180633

Fax - +421 556323173

SUPPLEMENTARY FIGURES

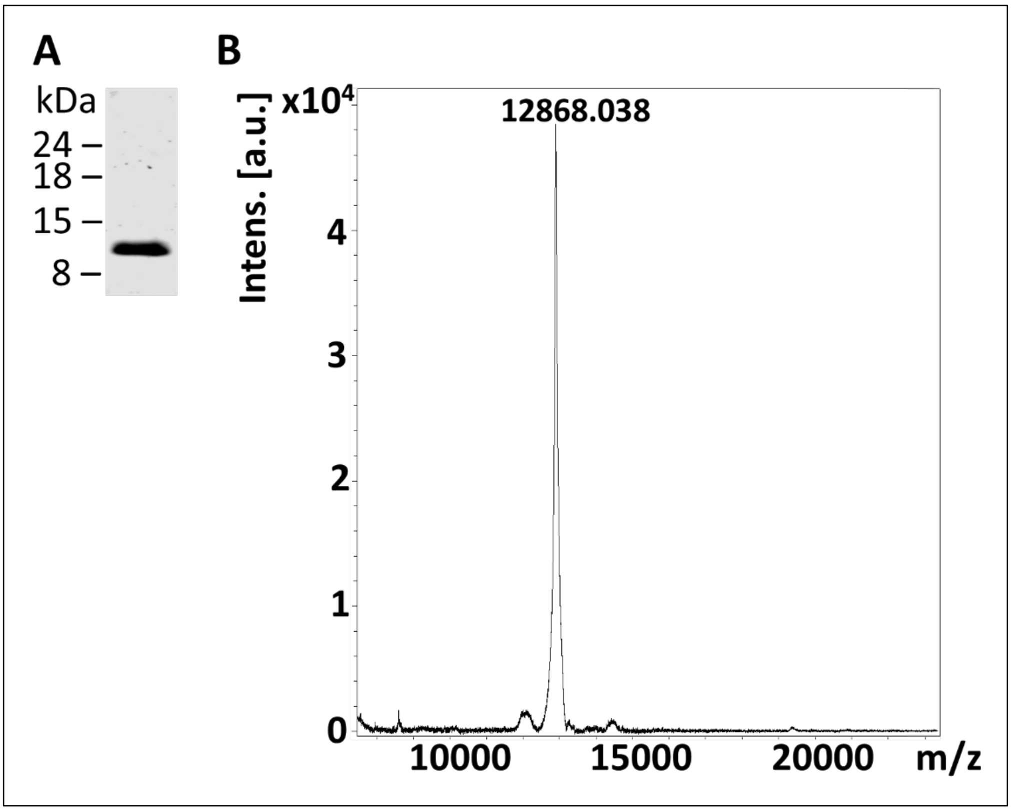

Supplementary Figure S1. Production of rDIII and quality control. *A – Purified rDIII separated with LDS-PAGE; B – Molecular mass of rDIII confirmed by MALDI-TOF MS. The observed mass of rDIII is [M + H]+. The observed molecular weight of rDIII WNV (12868.038 Da) was consistent with the theoretical molecular mass predicted using Geneious Pro v9.1.8 software (12868 Da).*

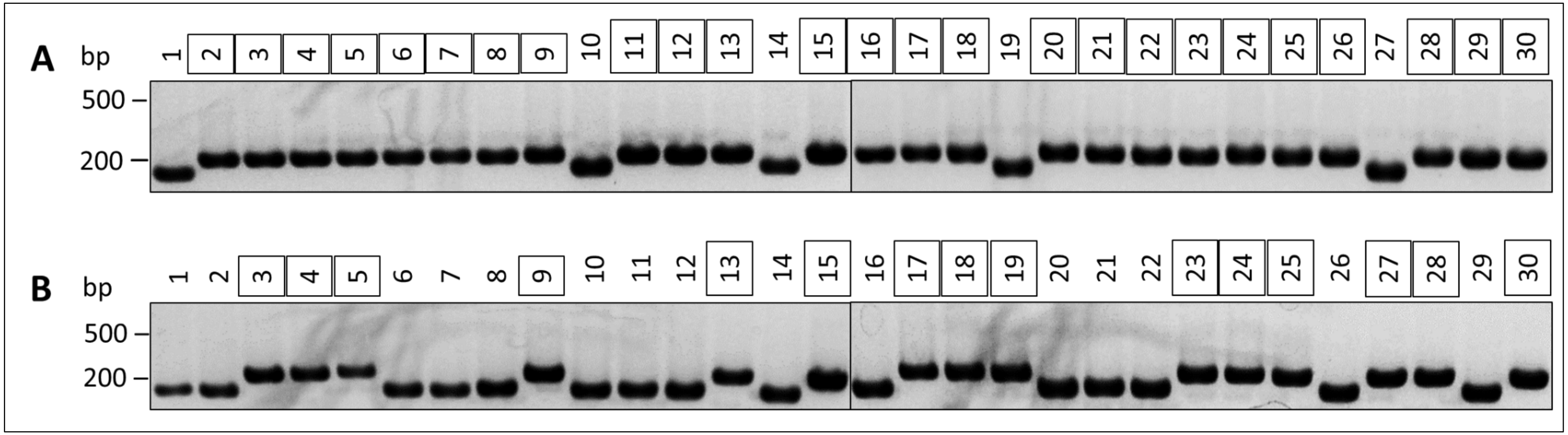

Supplementary Figure S2. Amplicons encoding 7-mer cylic or 12-mer linear peptides (from individual phage clones) that bind to rDIII. *ssDNA from 30 randomly picked phage plaques was isolated and fragments encoding 7-mer cyclic or 12-mer linear peptides were PCR amplified using vector-specific primers* (**Supplementary Table S1**)*. A – amplicons of C7C-mer phage clones (1 – 30); B – amplicons of 12-mer phage clones (1 – 30). Framed amplicons were selected for DNA sequencing. Please note that, unframed amplicons had lower molecular weight and contain no peptide sequence (the sequence does not have insert). Such amplicons were not sequenced.*

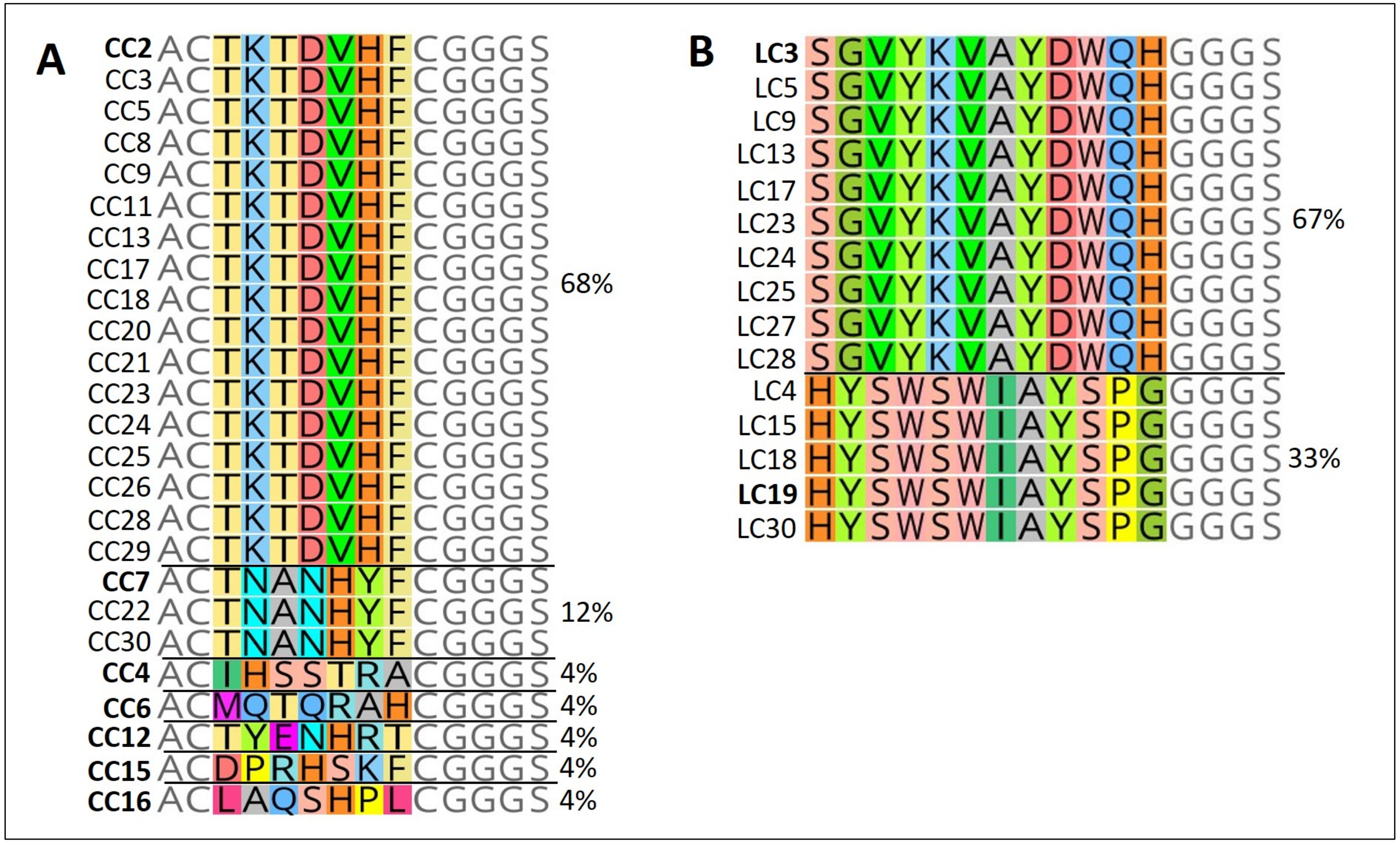

Supplementary Figure S3. Alignment of 7-mer cyclic and 12-mer peptide sequences selected after the last round of panning. *Sequence alignment (in Geneious Pro v9.1.8) of individual phage clones bearing 7-mer cyclic (A) or 12-mer linear (B) peptides that bind to rDIII. A representative phage clone (in bold) from each group was chosen for overexpression of the peptide in E. coli. The percentage represents a frequency of appearance. CC – phage clone carrying 7-mer cyclic peptide; LC – phage clone carrying 12-mer linear peptide.*

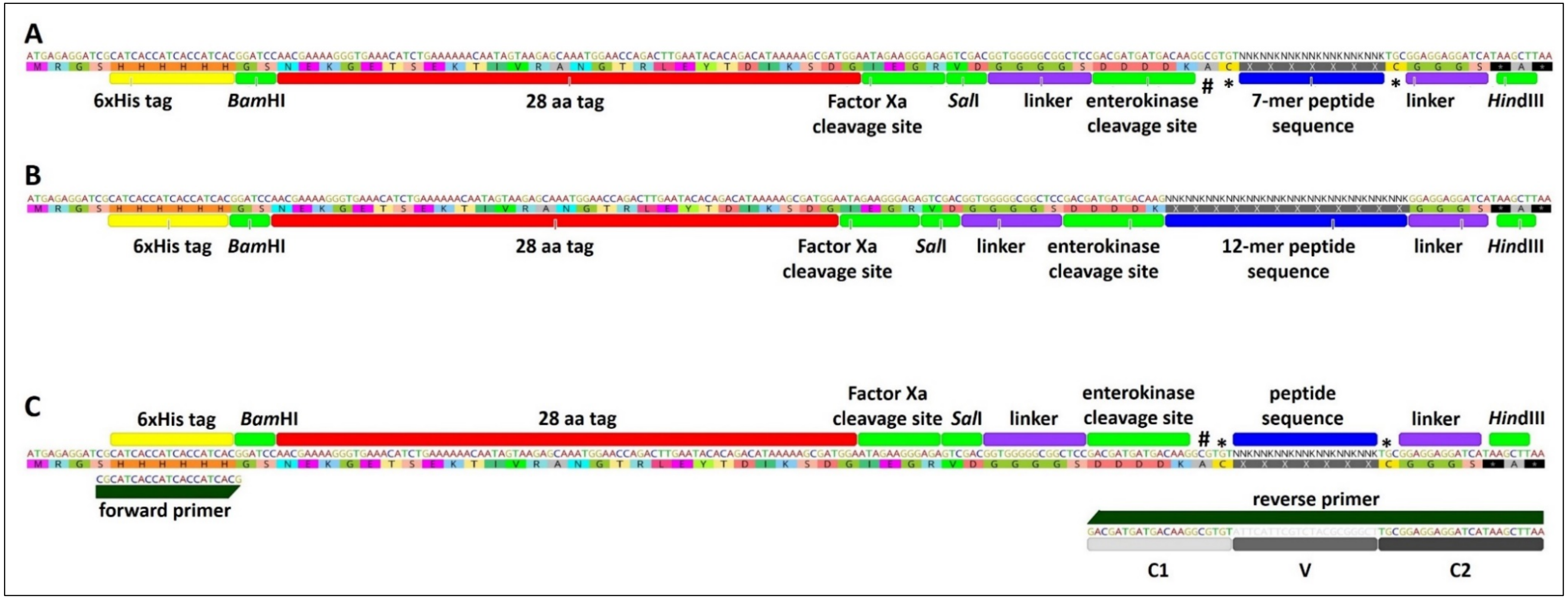

Supplementary Figure S4. The expression cassette used to produce 7-mer or 12-mer peptides. *The expression cassette was ligated into pQE-30-UA-mCherry vector and electroporated into E. coli SHuffle T5 strain. The expression cassette was designed using Geneious Pro v9.1.8. A – protein construct containing 7-mer peptide sequence; B – protein construct with incorporated 12-mer peptide sequence. C – schematic representation of the PCR. C1 – the conserved segment in the reverse primer complementary to the enterokinase cleavage site; V – variable part in the reverse primer encoding the 7-mer or 12-mer peptide sequence; C2 – the conserve region in the reverse primer complementary to GGGS linker and HindIII restriction site; "#" – codon for alanine (please note that, alanine was incorporated in protein construct as a part of a peptide based on the manufacturer's instructions (New England Biolabs); "*" – codons for cysteines flanking 7-mer peptide sequence.*

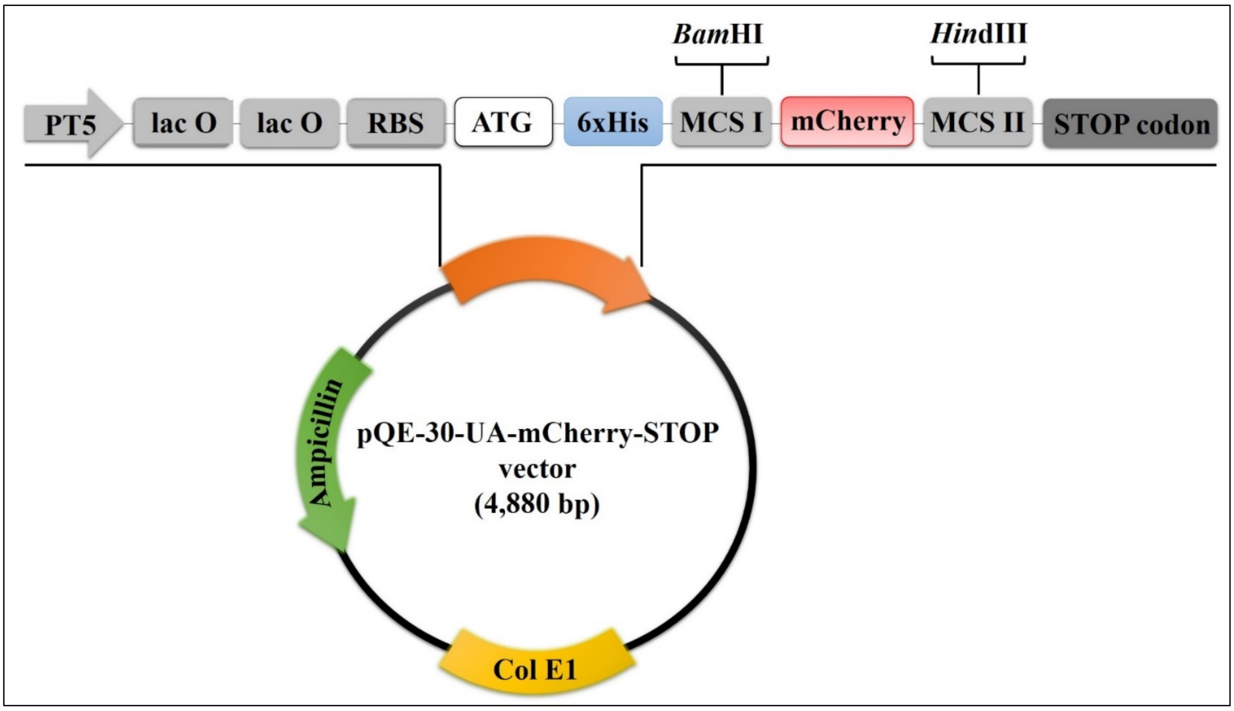

Supplementary Figure S5. pQE-30-UA-mCherry-STOP expression vector. *Col E1 – origin of replication; ampicillin – beta lactamase gene; PT5 – T5 promoter; lac O – lac operon; RBS – ribosome binding site; ATG – start codon; 6xHis tag; MCS I/MCS II – multiple cloning sites; mCherry – red fluorescent protein that serves as stuffer; stop codon. Please note that, mentioned restriction enzymesin MCS were used in the study.*

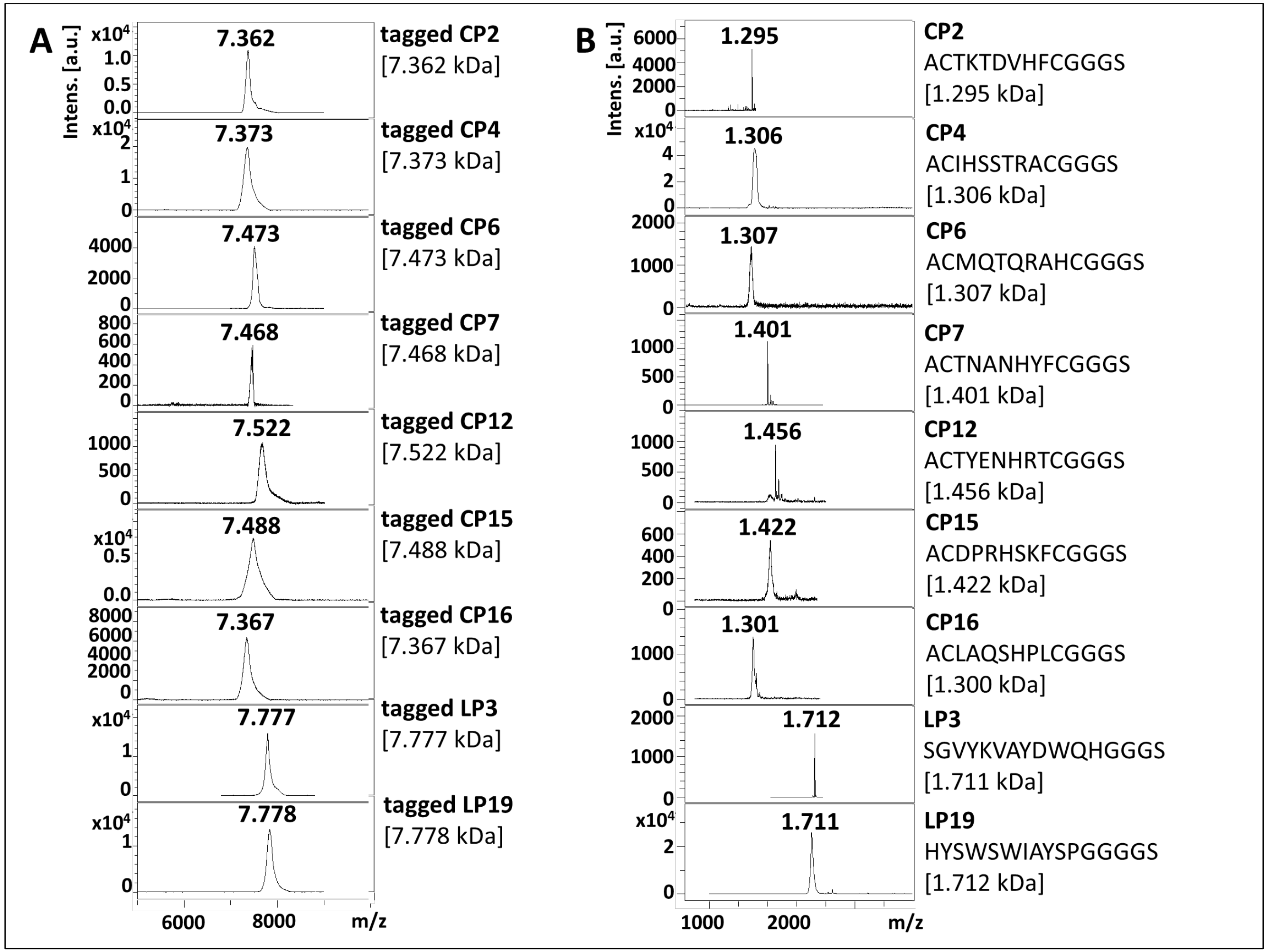

Supplementary Figure S6. Confirmation of the molecular masses of peptides by MALDI-TOF MS. *A – molecular masses of tagged peptides; B – molecular masses of peptides (without the tag) after removal of the tag (6xHis tag – 28 aa tag – GGGGS linker). Predicted molecular weight by Genious Pro v9.1.8 software is framed. CP – 7-mer cyclic peptide; LP – 12-mer linear peptide.*

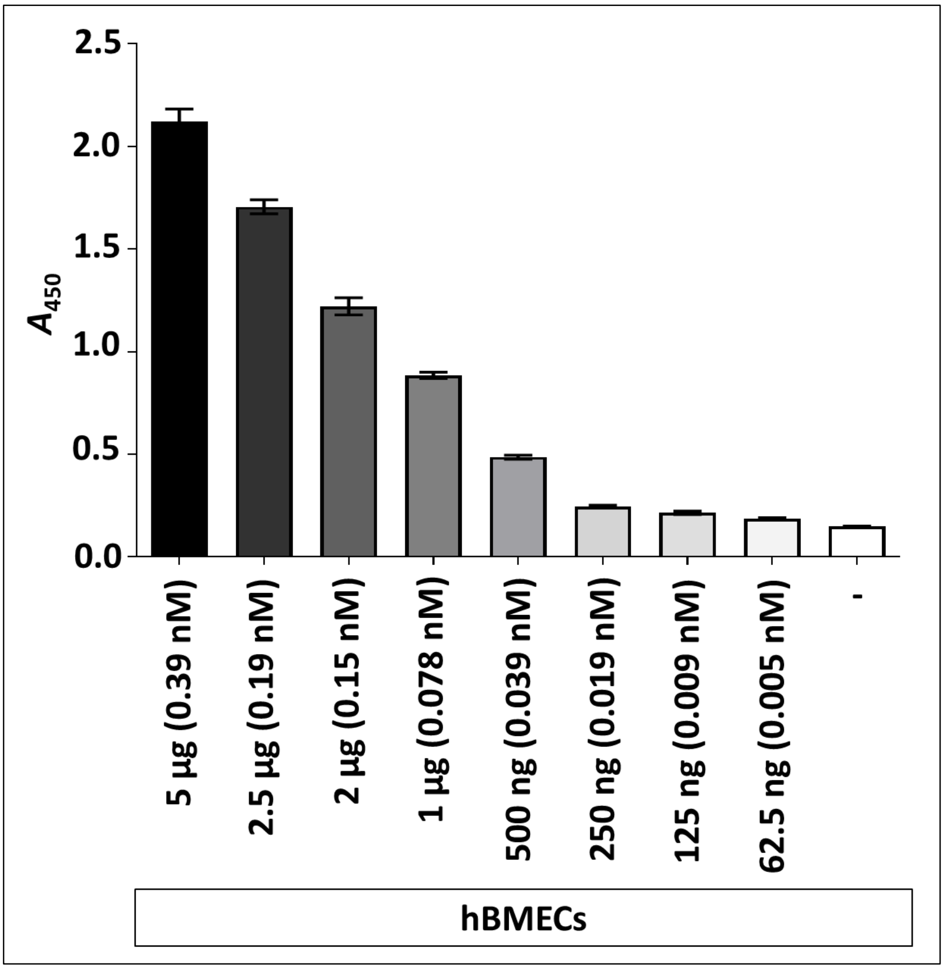

Supplementary Figure S7. Concentration-dependent interaction between rDIII and hBMECs proteins assessed by ELISA. *rDIII at various concentrations was incubated with hBMECs (coated in microtitre wells, in frame) to get the minimum concentration of rDIII sufficient to show its interaction with hBMECs proteins. Data present mean of triplicates with ± S.D. A – Absorbance; hBMECs – protein extract of hBMECs.*

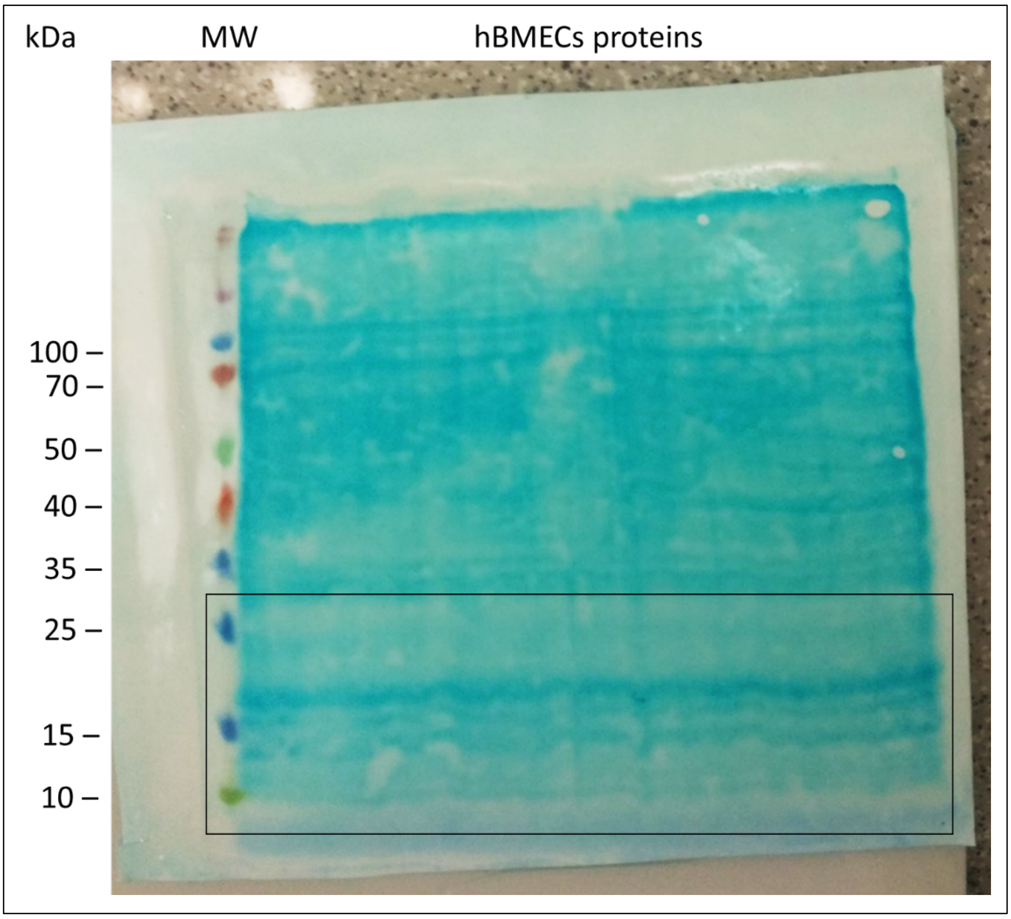

Supplementary Figure S8. Schematic representation of the low molecular weight proteins excision for Western blot. *The proteins extracted from hBMECs were separated by LDS-PAGE and transblotted on the NC membrane. The framed part of the NC membrane containing the low molecular weight hBMECs proteins (~25 kDa – ~10 kDa) was excised and sliced to obtain vertical strips (2mm thick), which were used in Western Blot to show ability of the peptides to block interaction between rDIII and the receptor of hBMECs. MW – molecular weight marker; hBMECs proteins – protein extract of hBMECs.*

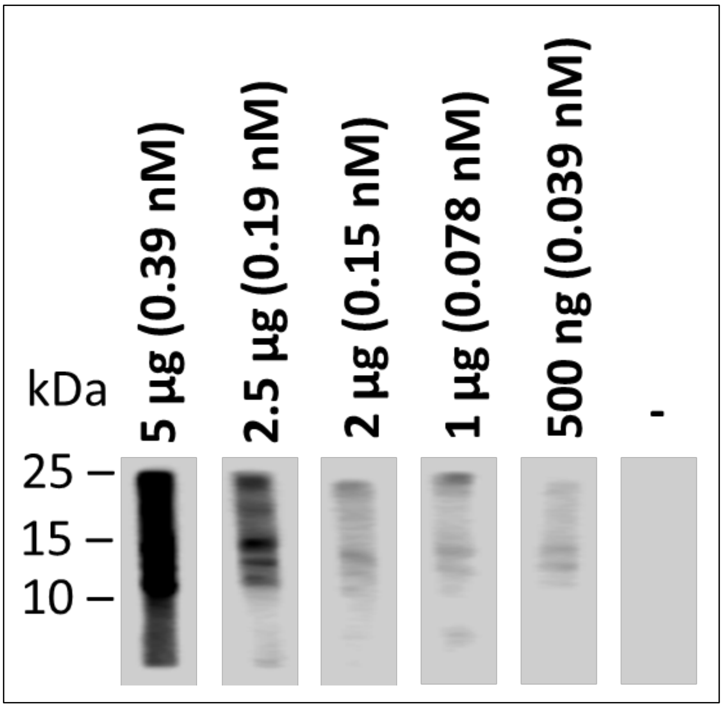

Supplementary Figure S9. Concentration-dependent interaction between low molecular weight proteins of hBMECs and rDIII checked by Western blotting. *rDIII at various concentrations was incubated with transblotted proteins of hBMECs on NC to get the minimum concentration of rDIII necessary to detect the interaction. In negative control (-), rDIII was omitted from the assay.*

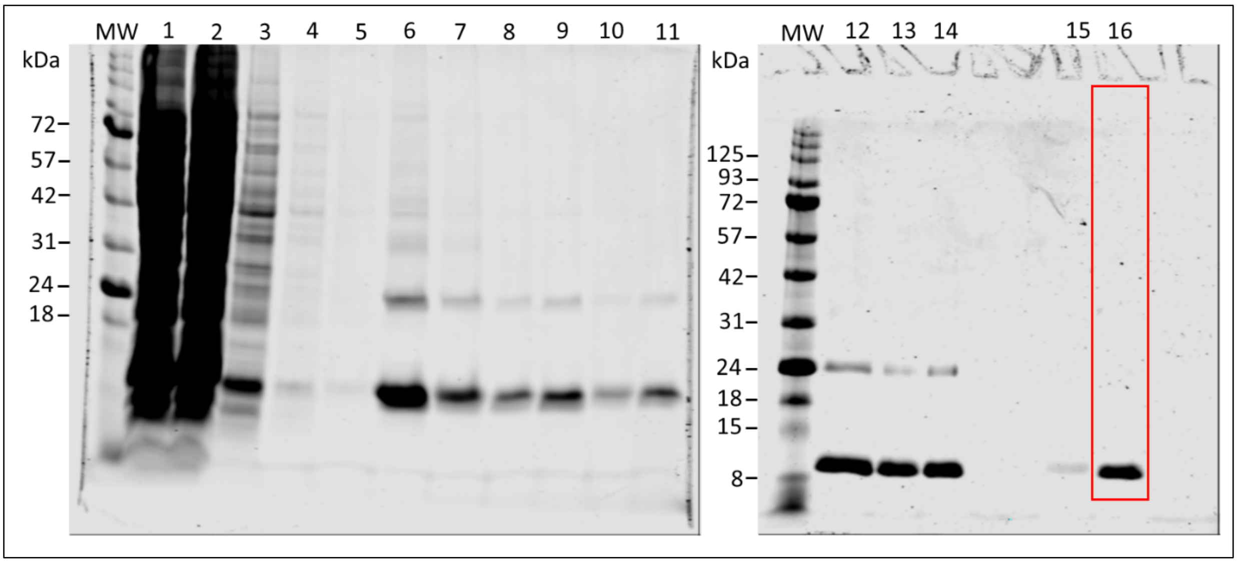

Supplementary Figure S10. The original figures of the gels used to make Supplementary Figure S1. *Purified rDIII separated on LDS-PAGE. MW – molecular weight marker; lane 1 – whole-cell lysate of E. coli overexpressing rDIII; lane 2 – flow-through obtained during nickel affinity chromatography; lane 3 – 1st washing of the nickel affinity beads after protein binding; lanes 4 and 5 – subsequent washing of the beads; lanes 6 - 11 – step wise elution performed with increasing concentrations of imidazole; lanes 12 - 13 – elute from lanes 6 to 11 after concentration; lanes 15 - 16 – rDIII eluted from MWCO gel filtration. The red box denotes the region of the original gel used in Supplementary Figure S1.*

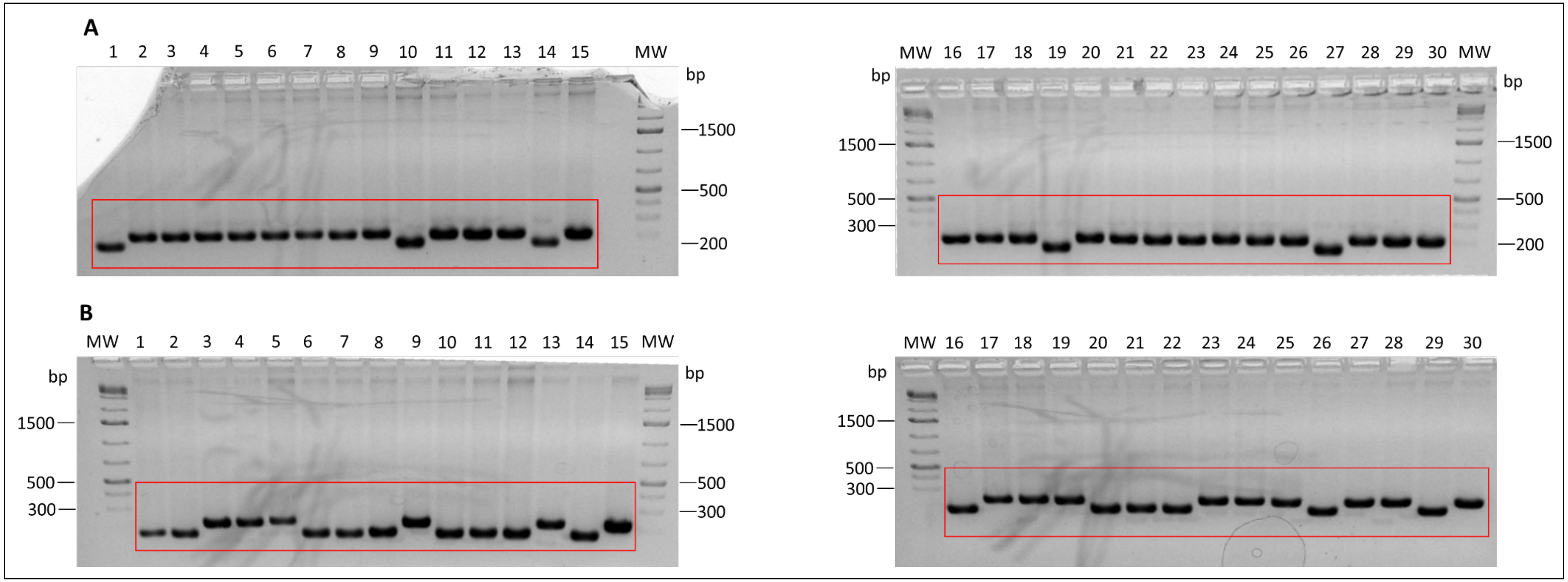

Supplementary Figure S11. The original figures of the gels used to make Supplementary Figure S2. *A – DNA agarose gel electrophoresis of dsDNA of randomly picked C7C-mer phage clones (1 – 30). B – DNA agarose gel electrophoresis of dsDNA of randomly picked 12-mer phage clones (1 – 30). MW – molecular weight marker. The red boxes denote the regions of the original gels used in Supplementary Figure S2.*

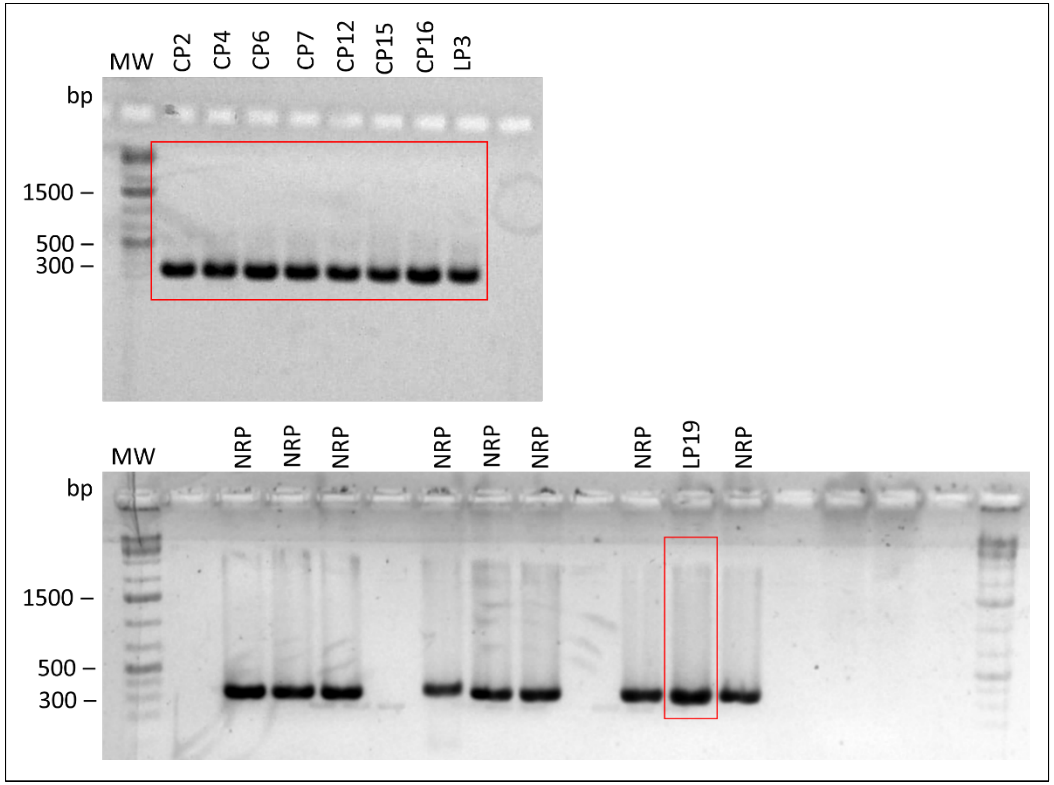

Supplementary Figure S12. The original figure of the gels used to make Panel A in Figure 2. *Agarose gel electrophoresis of the PCR amplicons confirming the correct insertion of DNA coding tagged DIII binding peptides in the transformants. CP – cyclic peptide; LP – linear peptide; NRP – results (proteins) not related to this manuscript; MW – molecular weight marker. The red boxes denote the regions of the original gels used in Figure 2A.*

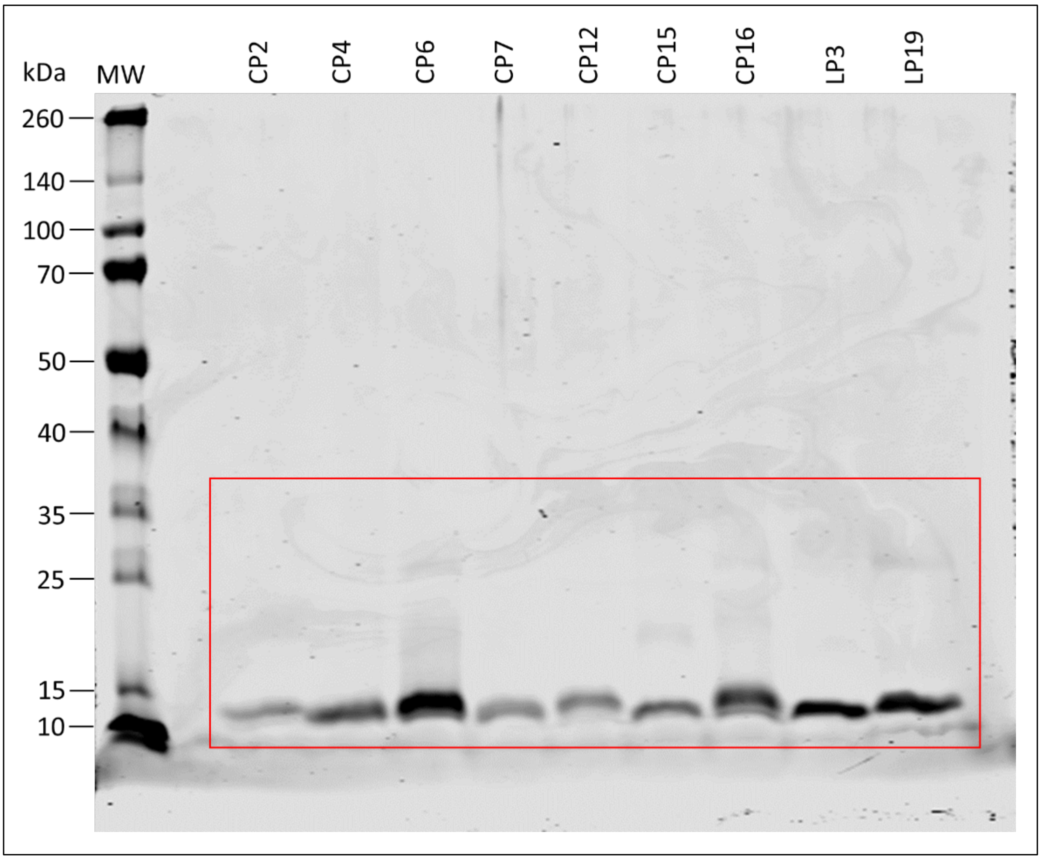

Supplementary Figure S13. The original figure of the gel used to make Panel B in Figure 2. *The purified tagged peptides were pre-incubated with 500 mM DTT for 10 min at 70°C and subsequently separated on LDS-PAGE (300 ng/well). MW – molecular weight marker; CP – cyclic peptide; LP – linear peptide. The red box denotes the region of the original gel used in Figure 2B.*

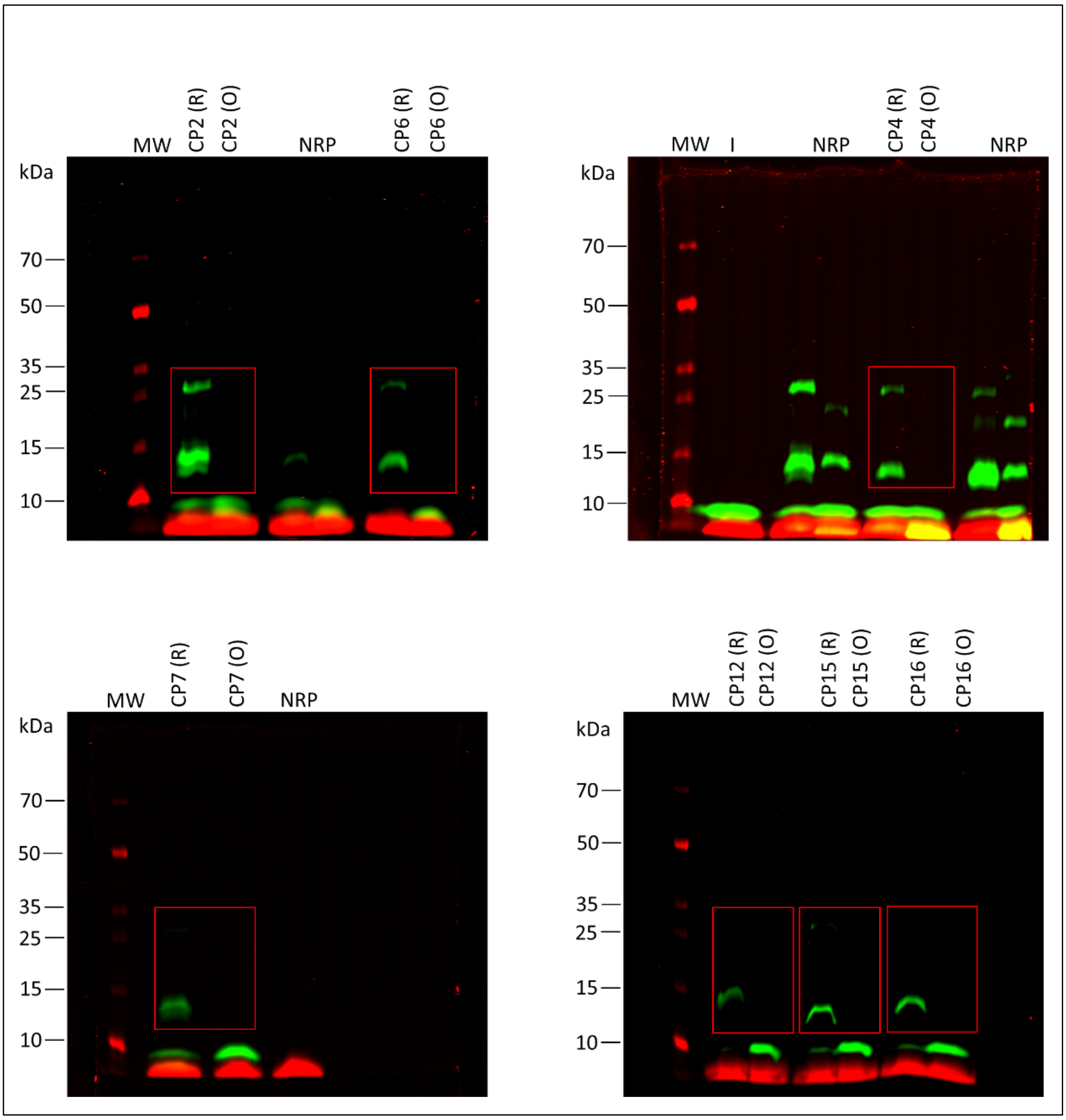

Supplementary Figure S14. The original figures of the gels used to make Panel E in Figure 2. *MW – molecular weight marker; CP – cyclic peptide; R – reduced form of tagged peptide; O – oxidized form of tagged peptide; MW – molecular weight marker; NRP – results (proteins) not related to this manuscript. The red boxes denote the regions of the original gels used in Figure 2E.*

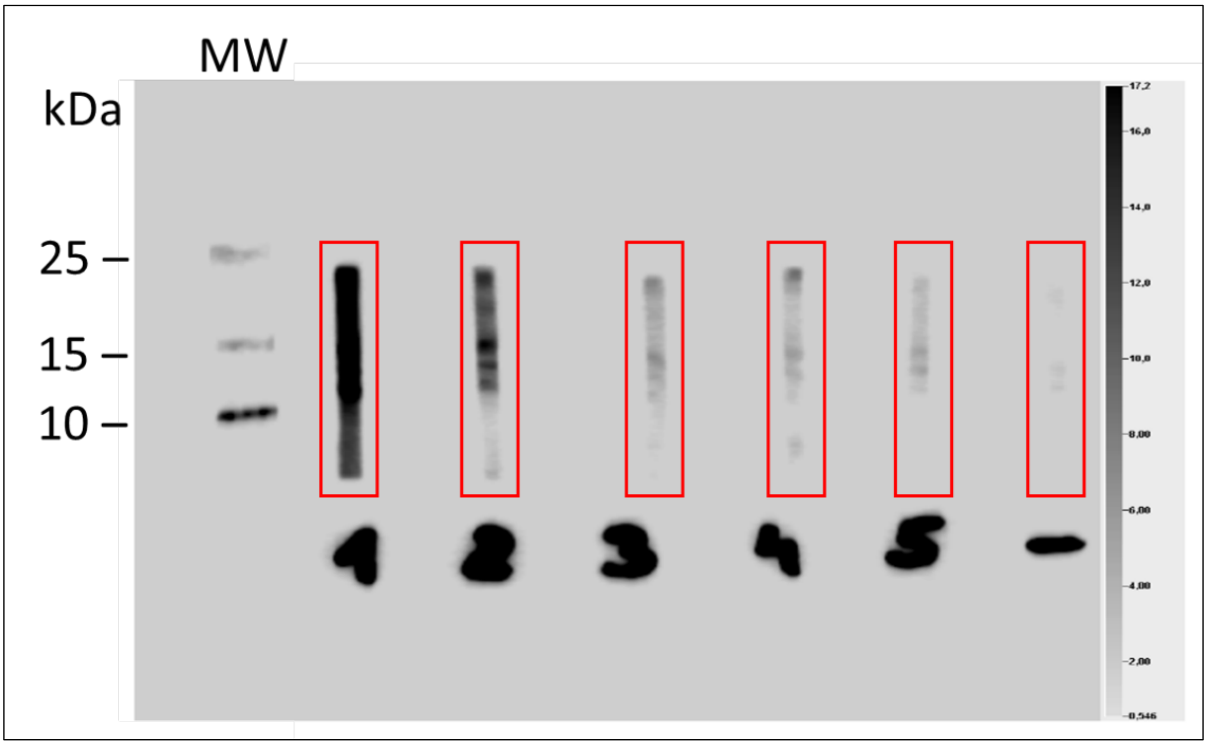

Supplementary Figure S15. The original figure of the blot used to make Supplementary Figure S9. *1 - 5 µg (0.39 nM); 2 – 2.5 µg (0.19 nM); 3 – 2 µg (0.15 nM); 4 – 1 µg (0.078 nM); 5 – 0.5 µg (0.039 nM); MW – molecular weight marker. The red boxes denote the regions of the original blot used in Figure S9.*

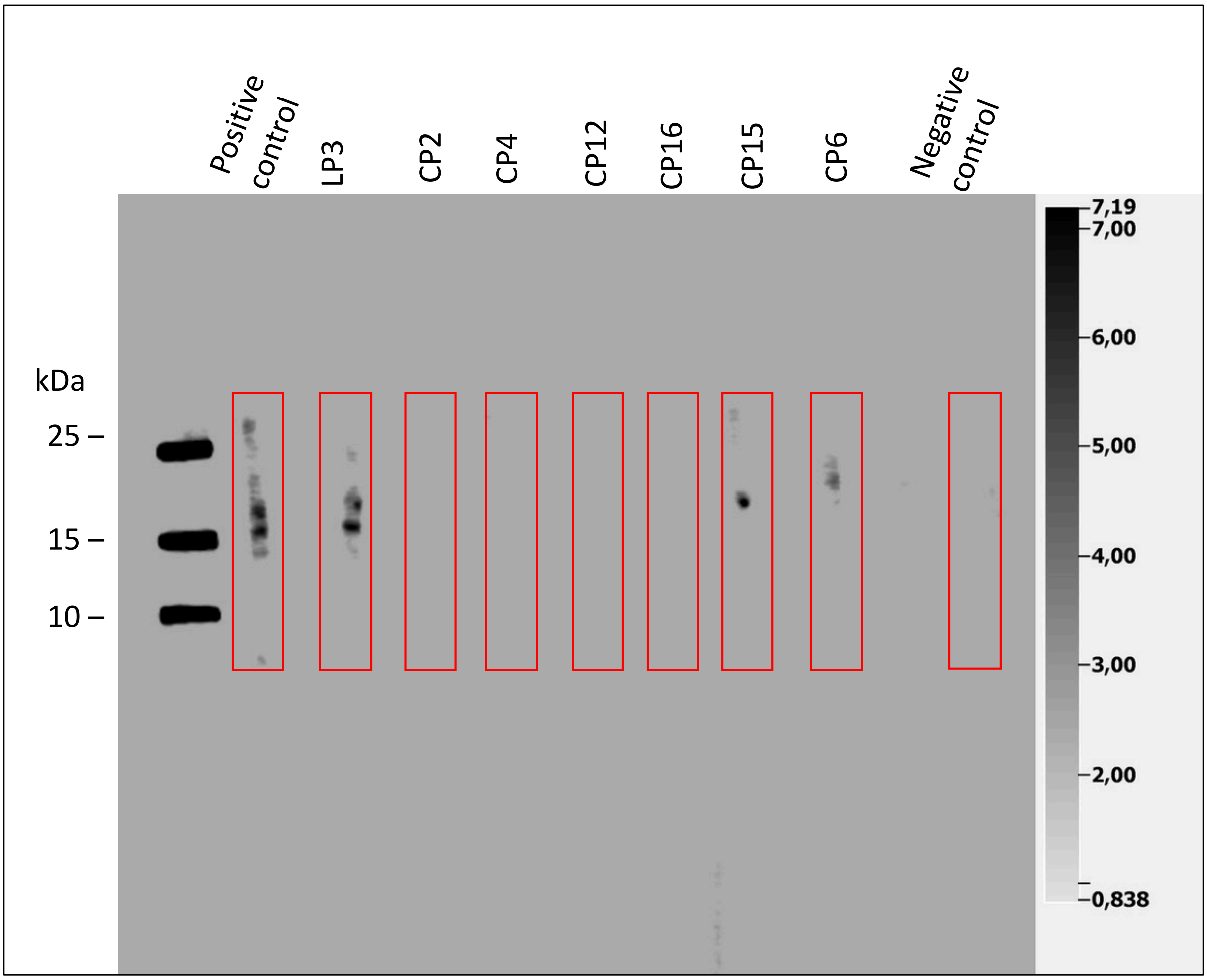

Supplementary Figure S16. The original figure of the blot used to make Panel B in Figure 4. *The interaction was detected with HisProbe-HRP conjugate and chemiluminescent substrate. Please note that each peptide was pre-incubated with rDIII and allowed to interact with hBMECs proteins. MW – molecular weight marker; positive control (non-treated rDIII incubated with the NC strip transblotted with protein extract of hBMECs); Negative control (the NC strip transblotted with protein extract of hBMECs incubated with TBS); CP – cyclic peptide; LP – linear peptide; MW – molecular weight marker. The red boxes denote the regions of the original blot used in Figure 4B. Please note that, blots with transferred hBMECs proteins were cut into strips (2mm thick, please see Supplementary Figure S8) prior to incubation with preblocked rDIII. The edges of the blots (strips) were not be visible after capturing the signal.*

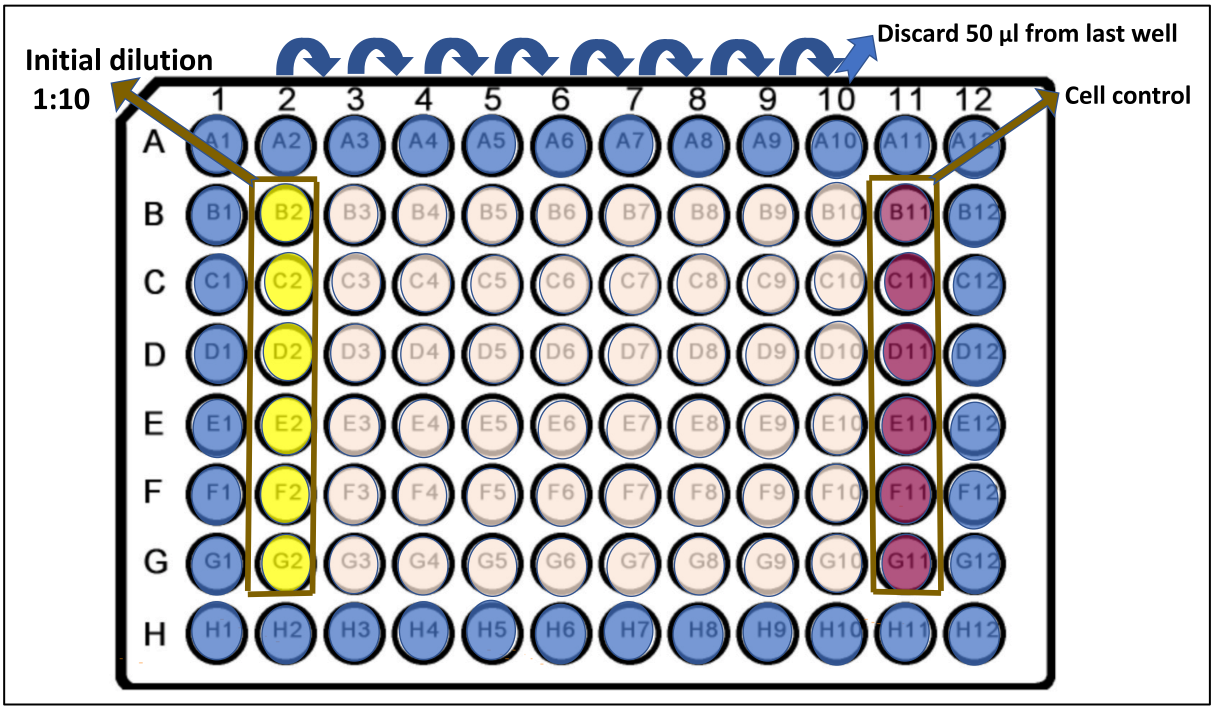

Supplementary Figure S17. 96-well plate template for VLP titration. *Dilution plate: the blue wells in the periphery indicate wells in which 250 µL of sterilized water was added (no samples). The maroon wells (B11 – G11) show the cell control (CC) wells, in which only DMEM (150 µL) was added. Yellow wells were used for the initial dilution of VLP (1:10) and wells in column 3 to 10 were used for serial dilution as follows: in B2 to G2 well, 135 µL of complete DMEM was added, while in remaining wells (B3 – G11) 100 µL of complete DMEM was added. 15 µL of pseudotyped virus was added in each well in column 2 (wells B2 – G2). Mixed six times. 50 µL from the wells B2 – G2 were transferred to wells B3 – G3 with a multi-channel pipette, mixed six times and 50 µL were transferred to B4 – G4 wells. Dilution was repeated till the B10–G10 wells. Finally, 50 µL of the solution from each well from column 10 was discarded.*

HEK293T cells (20,000 cells/well) were cultured in 96-well plate (except peripheral wells) in complete DMEM medium overnight. Medium was removed carefully and content of each well from dilution plate was transferred to the culture plate. Plate was incubated at 37 °C, 5% CO_2, for 48 h. After incubation, wells were checked under the microscope and subjected for luciferase assay.

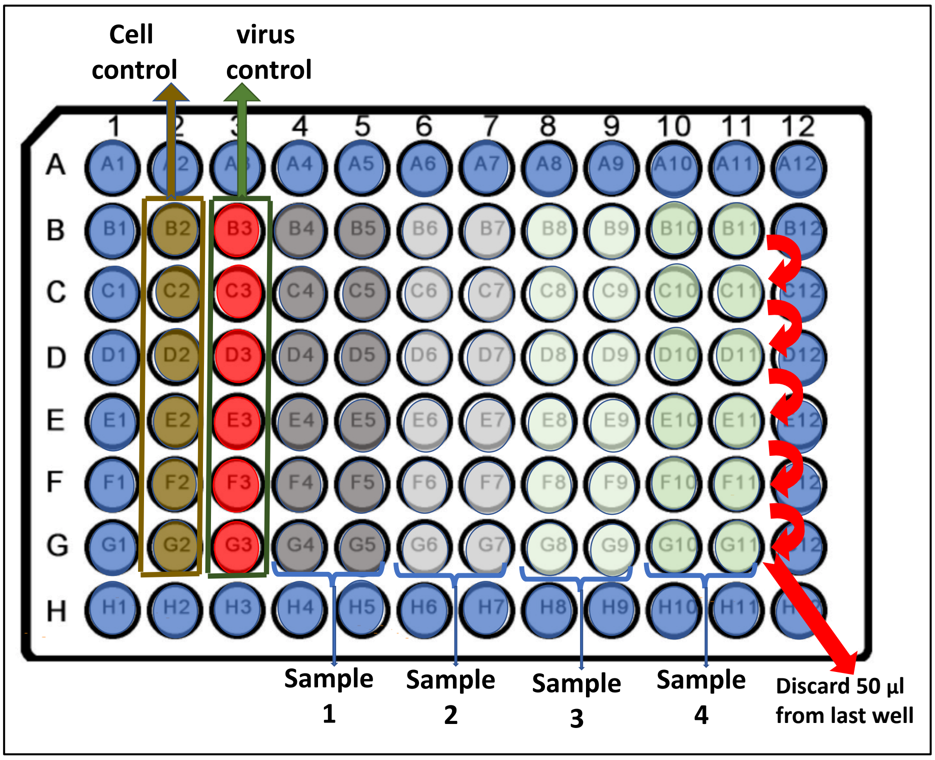

Supplementary Figure S18. Neutralization assay. ***Dilution plate:*** *the blue wells in the periphery indicate wells in which 250 µL of sterilized water was added (no samples). The dark-brown wells (B2 – G2) show the cell control wells, in which only DMEM (150 µL) was added. Red wells (B3 – G3) indicate virus control wells, in which 100 µL of DMEM and 50 µL of VLP was added.* ***Neutralization:*** *Column 4 and 5 are for one sample (duplicate), thus 4 samples can be tested in one plate as shown in figure. In wells C4 – G11, 100 µL of DMEM was added (not in row B, B4-B11). Peptides were serially diluted as follows in dilution plate: 1 µg of the peptide (in 7.5 µL) was mixed with 142.5 µL of DMEM in wells B4 and B5. Content was mixed six times. Fifty microliters from each well were transferred to C4 and C5, mixed six times and 50 µL from C4 and C5 were transferred to D4 and D5. Dilution was continued till G4 and G5 wells and 50 µL from those wells were discarded. 50 µL of VLP (diluted to achieve 400-500 $TCID_{50}$/well) were added in each well in duplicate. Plate was incubated for 3 hrs at room temperature.* ***Transfter of peptide and VLP mix to cells:*** *HEK293T cells (20,000 cells/well) were cultured in 96-well plate (except peripheral wells) in complete DMEM medium overnight. Medium was removed carefully and content of each well from dilution plate was transferred to the culture plate. Plate was incubated at 37 °C, 5% CO_2, for 48 h. After incubation, wells were checked under the microscope and subjected for luciferase assay.*

SUPPLEMENTARY TABLES

Supplementary Table S1. Primers and PCR conditions used in this study

**Protein/Peptide**	**Primer**	**Sequence (5´ – 3´)**	**Amplicon length (bp)**
Primers used to amplify DNA encoding 7-mer and 12-mer phage displayed-peptides for subsequent sequencing			
-	M13KE sense	TCGCAATTCCTTTAGTGGTACCTTTC	-
-	M13KE antisense	GCATTCCACAGACAGCCCTCA	
**Reaction mix:** PCR-grade water – 38.1 µL; 10X Taq Polymerase Buffer – 5 µL; 12.5 mM dNTP – 0.5 µL; 10 µM forward primer – 0.6 µL; 10 µM reverse primer – 0.6 µL; 5 units/µL Taq polymerase – 0.2 µL; DNA – 5 µL<br>**Program:** 94°C – 2 min; 35 x [94°C – 30 sec, 65°C – 30 sec, 72°C – 1 min 45 sec]; 72°C – 10 min; 4°C – forever			
Primers used for the production of promising anti-WNV blocking peptides			
cyclic peptide 2 (CP2)	UA-Insertom-His sense	CGCATCACCATCACCATCACG	198
	antisense	CTTAAGCTTATGATCCTCCTCCGCAAAAATGAACATCCGTCTTAGTACACGCCTTGTCATCATCGTC	
cyclic petide 4 (CP4)	UA-Insertom-His sense	CGCATCACCATCACCATCACG	198
	antisense	CTTAAGCTTATGATCCTCCTCCGCAAGCCCGCGTAGACGAATGAATACACGCCTTGTCATCATCGTC	
cyclic petide 6 (CP6)	UA-Insertom-His sense	CGCATCACCATCACCATCACG	198
	antisense	CTTAAGCTTATGATCCTCCTCCGCAATGAGCCCTCTGAGTCTGCATACACGCCTTGTCATCATCGTC	
cyclic petide 7 (CP7)	UA-Insertom-His sense	CGCATCACCATCACCATCACG	198
	antisense	CTTAAGCTTATGATCCTCCTCCGCAAAAATAATGATTAGCATTAGTACACGCCTTGTCATCATCGTC	

cyclic petide 12 (CP12)	UA-Insertom-His sense	CGCATCACCATCACCATCACG	198
	antisense	CTTAAGCTTATGATCCTCCTCCGCAAGTCCTATGATTCTCATACGTACACGCCTTGTCATCATCGTC	
cyclic petide 15 (CP15)	UA-Insertom-His sense	CGCATCACCATCACCATCACG	198
	antisense	CTTAAGCTTATGATCCTCCTCCGCAAAACTTACTATGACGAGGATCACACGCCTTGTCATCATCGTC	
cyclic petide 16 (CP16)	UA-Insertom-His sense	CGCATCACCATCACCATCACG	198
	antisense	CTTAAGCTTATGATCCTCCTCCGCACAGCGGATGCGACTGCGCCAAACACGCCTTGTCATCATCGTC	
linear peptide 3 (LP3)	UA-Insertom-His sense	CGCATCACCATCACCATCACG	204
	antisense	CTTAAGCTTATGATCCTCCTCCATGCTGCCAATCATACGCAACCTTATACACACCACTCTTGTCATCATCGTCGGAGCC	
linear peptide 19 (LP19)	UA-Insertom-His sense	CGCATCACCATCACCATCACG	204
	antisense	CTTAAGCTTATGATCCTCCTCCTCCAGGCGAATAAGCAATCCAAGACCAAGAATAATGCTTGTCATCATCGTCGGAGCC	
Restriction sites are depicted with underlined nucleotides. AAGCTT – *Hind*III<br>**Reaction mix:** PCR-grade water – 38.1 µL; 10X Taq Polymerase Buffer – 5 µL; 12.5 mM dNTP – 0.5 µL; 10 µM forward primer – 0.6 µL; 10 µM reverse primer – 0.6 µL; 5 units/µL Taq polymerase – 0.2 µL; DNA – 5 µL<br>**Program:** 94°C – 2 min; 35 x [94°C – 30 sec, 65°C – 30 sec, 72°C – 1 min 45 sec]; 72°C – 10 min; 4°C – forever			
Primers used to confirm the presence of the insert in transformants			
-	UA-Insertom-His sense	CGCATCACCATCACCATCACG	-
-	UA-Insertom-GFP antisense	ACCAAATTGGGACAACACCAGTG	
**Reaction mix**: PCR-grade water – 38.1 µL; 10X Taq Polymerase Buffer – 5 µL; 12.5 mM dNTP – 0.5 µL; 10 µM forward primer – 0.6 µL; 10 µM reverse primer – 0.6 µL; 5 units/µL Taq polymerase – 0.2 µL; DNA – 5 µL<br>**Program:** 94°C – 2 min; 35 x [94°C – 30 sec, 65°C – 30 sec, 72°C – 60 sec]; 72°C – 10 min; 4°C – forever			

Supplementary Table S2. Nucleotide sequences of fragments used for ligation into the pQE-30-UA-mCherry-STOP expression vector for production of tagged peptides

Protein	Nucleotide sequence
cyclic peptide 2 (CP2)	**CGCATCACCATCACCATCACG**GATCCAACGAAAAGGGTGAAACATCTGAAAAAACAATAGTAAGAGCAAATGGAACCAGACTTGAATACACAGACATAAAAAGCGATGGAATAGAAGGGAGAGTCGACGGTGGGGGCGGCTCC**GACGATGATGACAAGGCGTGTACTAAGACGGATGTTCATTTTTGCGGAGGAGGATCAT**
cyclic peptide 4 (CP4)	**CGCATCACCATCACCATCACG**GATCCAACGAAAAGGGTGAAACATCTGAAAAAACAATAGTAAGAGCAAATGGAACCAGACTTGAATACACAGACATAAAAAGCGATGGAATAGAAGGGAGAGTCGACGGTGGGGGCGGCTCC**GACGATGATGACAAGGCGTGTATTCATTCGTCTACGCGGGCTTGCGGAGGAGGATCAT**
cyclic peptide 6 (CP6)	**CGCATCACCATCACCATCACG**GATCCAACGAAAAGGGTGAAACATCTGAAAAAACAATAGTAAGAGCAAATGGAACCAGACTTGAATACACAGACATAAAAAGCGATGGAATAGAAGGGAGAGTCGACGGTGGGGGCGGCTCC**GACGATGATGACAAGGCGTGTATGCAGACTCAGAGGGCTCATTGCGGAGGAGGATCAT**
cyclic peptide 7 (CP7)	**CGCATCACCATCACCATCACG**GATCCAACGAAAAGGGTGAAACATCTGAAAAAACAATAGTAAGAGCAAATGGAACCAGACTTGAATACACAGACATAAAAAGCGATGGAATAGAAGGGAGAGTCGACGGTGGGGGCGGCTCC**GACGATGATGACAAGGCGTGTACTAATGCTAATCATTATTTTTGCGGAGGAGGATCAT**
cyclic peptide 12 (CP12)	**CGCATCACCATCACCATCACG**GATCCAACGAAAAGGGTGAAACATCTGAAAAAACAATAGTAAGAGCAAATGGAACCAGACTTGAATACACAGACATAAAAAGCGATGGAATAGAAGGGAGAGTCGACGGTGGGGGCGGCTCC**GACGATGATGACAAGGCGTGTACGTATGAGAATCATAGGACTTGCGGAGGAGGATCAT**

cyclic peptide 15<br>(CP15)	**CGCATCACCATCACCATCACG**GATCCAACGAAAAGGGTGAAACATCTGAAAAAACAATAGTAAGAGCAAATGGAACCAGACTTGAATACACAGACATAAAAAGCGATGGAATAGAAGGGAGAGTCGACGGTGGGGGCGGCTCC**GACGATGATGACAAGGCGTGTGATCCTCGTCATAGTAAGTTTTGCGGAGGAGGATCAT**
cyclic peptide 16<br>(CP16)	**CGCATCACCATCACCATCACG**GATCCAACGAAAAGGGTGAAACATCTGAAAAAACAATAGTAAGAGCAAATGGAACCAGACTTGAATACACAGACATAAAAAGCGATGGAATAGAAGGGAGAGTCGACGGTGGGGGCGGCTCC**GACGATGATGACAAGGCGTGTTTGGCGCAGTCGCATCCGCTGTGCGGAGGAGGATCAT**
linear peptide 3<br>(LP3)	**CGCATCACCATCACCATCACG**GATCCAACGAAAAGGGTGAAACATCTGAAAAAACAATAGTAAGAGCAAATGGAACCAGACTTGAATACACAGACATAAAAAGCGATGGAATAGAAGGGAGAGTCGACGGTGGGGGCGGCTCC**GACGATGATGACAAGAGTGGTGTGTATAAGGTTGCGTATGATTGGCAGCATGGAGGAGGATCAT**
linear peptide 19<br>(LP19)	**CGCATCACCATCACCATCACG**GATCCAACGAAAAGGGTGAAACATCTGAAAAAACAATAGTAAGAGCAAATGGAACCAGACTTGAATACACAGACATAAAAAGCGATGGAATAGAAGGGAGAGTCGACGGTGGGGGCGGCTCC**GACGATGATGACAAGCATTATTCTTGGTCTTGGATTGCTTATTCGCCTGGAGGAGGAGGATCAT**

Sense and antisense primer used for amplification are indicated in bold.

SUPPLEMENTARY METHODS

Supplementary Method S1

Culture of human brain microvascular endothelial cells (hBMECs). In brief, hBMECs (D3 cell line; Merck/Millipore, Czech Republic) were cultured in a 25 cm^2 cell culture flask coated with collagen type I (Sigma, USA) in EBM-2 medium (Lonza, UK) containing 10% FBS, 1.4 µM hydrocortisone (Sigma), 5 µg/mL ascorbic acid, 10 mM HEPES and 1 ng/mL bFGF (Sigma). Cells were incubated at 37°C in a humid atmosphere of 5% CO_2 until confluence.

Supplementary Method S2

Isolation of proteins from hBMECs. In short, the confluent monolayer of hBMECs was washed twice with PBS (pH 7.4). Cells were scraped with 1 mL of lysis solution (20 mM CHAPS, 300 mM NaCl, 50 mM HEPES, 0.1% sodium azide, and 1X proteases inhibitors; Sigma) and incubated 20 min on ice. After incubation, cells were disrupted with 5 cycles of sonication on ice (100% amplitude, 30 s). Debris was removed by centrifugation (10,000 × *g*, 5 min, 4°C) and protein fraction was subjected for gel filtration (Sephadex G-25, 30 ml column, *in-house* prepared) against PBS (pH 7.4) on ÄKTApurifier (2 mL/min flow, max 0.45 MPa pressure; GE-Healthcare, USA). Protein concentration was measured with Bradford assay and proteins were stored at -80°C till use.

Supplementary Method S3

Phage ELISA to assess the binding ability of eluted phages from the last round of panning to rDIII. rDIII (300 ng/well) diluted in TBS (pH 7.2) was incubated in Pierce Nickel Coated Plate (Thermo Fisher Scientific, Slovakia) overnight at 4°C. Unbound rDIII was washed with TBS containing 0.1% Tween 20 (0.1% TBST) and blocking of non-specific binding sites was

performed with 1% BSA in 0.1% TBST for 1 h at room temperature. After 3 washings with 0.1% TBST, amplified and purified phages (2 × 10^9 plaque-forming units, PFU) resuspended in 0.1% TBST containing 1% BSA were added and incubated for 1 h at room temperature with constant shaking. Wells were washed with 0.1% TBST and incubated with mouse anti-M13 monoclonal antibody (1:1,000 dilution in 0.1% TBST containing 1% BSA; GE Healthcare) for 1 h at room temperature with constant shaking. After three washings with 0.1% TBST, a recombinant Protein A/G peroxidase conjugate (1:10,000 dilution in 0.1% TBST with 1% BSA; Thermo Fisher Scientific) was added and incubated for 1 h at room temperature with constant shaking. After six washings, the chromogenic reaction was developed using a 1-Step Ultra TMB-ELISA substrate (Thermo Fisher Scientific). The reaction was stopped after 25 min with 2 M H_2SO_4 and absorbance (*A*) was measured at 450 nm. For negative controls, either rDIII or phages or anti-M13 antibody were excluded from the experiment. The assay was performed in triplicates.

Supplementary Method S4

Phage ELISA to assess the binding ability of individual phage clones to rDIII. The phage ELISA confirming the interaction between rDIII and individual phage clones was performed as described in **Supplementary Method S3**. In this case, 10^{11} PFU/clone/well were used in ELISA.

Supplementary Method S5

Production of tagged 7-mer cyclic and 12-mer linear peptides: preparation of expression cassette, clonal selection, and overexpression. A template of expression cassette was prepared as follows: 5′ – MRGS sequence (ATGAGAGGATCG) – 6xHis tag sequence (CATCACCATCACCATCAC) – *Bam*HI restriction site (GGATCC) – 84 bp sequence devoid of

cysteine and methionine codons (28 aa tag, part of $OspA_{127-154}$ protein; GenBank: JX274591.1) – Factor Xa cleavage site (ATAGAAGGGAGA) – *Sal*I restriction site (GTCGAC) – GGGGS linker (GGTGGGGGCGGCTCC) – enterokinase cleavage sequence (GACGATGATGACAAG) – [alanine codon (GCG)] – sequence encoding peptide with 7 aa (flanking with cysteine at both ends) or 12 aa – GGGS linker (GGCGGAGGTTCC) - stop codon (TAA) – *Hin*dIII restriction site (AAGCTT) – 3´ (schematically depicted in **Supplementary Fig. S4A, B**). To change the peptide sequence (7 aa or 12 aa) in the expression cassette, PCR was performed on the template presented above with a forward primer complementary to 6xHis tag sequence and reverse primer consisting of a variable sequence encoding 7 aa or 12 aa peptide. The example of PCR is presented in **Supplementary Fig. S4C.** The 3′ of each reverse primer was complementary to the enterokinase cleavage site and the 5´ was complementary to the GGGS linker and *Hin*dIII restriction site (**Supplementary Fig. S4**). Primers are presented in **Supplementary Table S1**.

PCR products were digested with restriction enzymes *Bam*HI and *Hin*dIII (Thermo Fisher Scientific) and ligated into an *in-house* modified pQE-30-UA-mCherry vector (**Supplementary Fig. S5**). Please note that in this vector mCherry serves as a stuffer sequence. The ligation mix was purified using NucleoSpin (Macherey-Nagel, Germany) and transformed into SHuffle T5 Express competent *E. coli* (New England Biolabs, USA). Transformants were selected from LB agar plates (Lysogeny broth; supplemented with 2% bacteriological agar, 1% glucose, and 100 μg/mL carbenicillin; Sigma). The presence of inserted gene in the transformants was confirmed by sequencing with vector-specific primers (**Supplementary Table S1**).

A single colony carrying inserted gene was cultivated in 2x TY medium (16 g/L tryptone, 10 g/L yeast extract, 5 g/L NaCl, pH 7.0) supplemented with 1% glucose, 100 μg/mL carbenicillin until OD_{600} = 0.6. Bacterial cells were pelleted (centrifugation at 17,880 × *g* for 15

min) and resuspended in a fresh 2x TY medium without glucose. Protein expression was induced with 0.5 mM IPTG (Fermentas, Slovakia) overnight at 25°C.

Supplementary Method S6

Production of tagged 7-mer cyclic and 12-mer linear peptides: purification of tagged peptides. After induction, cells were pelleted (17,880 × *g* for 15 min) and lysed in lysis buffer (0.03 M Na_2HPO_4, 0.5 M NaCl, 0.001% Tween 20, 10% glycerol, 1X protease inhibitor cocktail, Sigma) with four freeze-thaw cycles followed by sonication on ice (6 cycles; 30-s pulses, 100% amplitude). Tagged peptides were purified with nickel affinity chromatography as per the manufacturer´s instructions (Ni-NTA agarose beads; ABT agarose, Spain). Briefly, Ni-NTA beads were equilibrated with the buffer (50 mM Na_2HPO_4, 300 mM NaCl, 20 mM imidazole; pH 8.0). Lysate was incubated with beads for 1 h at 4°C with constant rotation (5 rpm). Unbound proteins were washed with washing buffer (50 mM Na_2HPO_4, 300 mM NaCl, 20 mM imidazole; pH 8.0) 4 times for 10 min. Tagged peptides were eluted in elution buffer (50 mM Na_2HPO_4, 300 mM NaCl, 250 mM imidazole; pH 7.0). The purified tagged peptides were immediately subjected to gel filtration (Sephadex G-25, 30 ml column, *in-house* prepared) against 20 mM ammonium bicarbonate buffer (pH 7.6) on ÄKTApurifier (2 ml/min flow, max 0.45 MPa pressure; GE-Healthcare) to remove imidazole and accomplish the buffer exchange. The purity of tagged peptides was evaluated with LDS-PAGE (**Supplementary Method S7**). Aliquots of purified tagged peptides were vacuum-dried and stored at room temperature until further use.

Supplementary Method S7

Production of tagged 7-mer cyclic and 12-mer linear peptides: quality assessment by LDS-PAGE. To reduce the disulfide bonds present in the tagged peptides, 300 ng of each tagged peptide were pre-incubated with 500 mM dithiothreitol (DTT; Sigma) with 4X lithium dodecyl sulfate sample buffer (4X LDS sample buffer; Invitrogen, Slovakia) for 10 min at 70°C. Electrophoresis was carried out at 30 mA in 1X running buffer (20X NuPAGE MOPS SDS running buffer, Invitrogen) until the dye reached the bottom of the gel (12% Bis-Tris polyacrylamide 12-well gel; Invitrogen). Tagged peptides were stained with Coomassie staining (Bio-Rad, USA) and scanned on the Odyssey CLx imaging system (LI-COR Biosciences, USA).

Supplementary Method S8

Production of tagged 7-mer cyclic and 12-mer linear peptides: quality assessment by MALDI-TOF MS. Samples (tagged peptides before and after enterokinase digestion) were desalted and concentrated with ZipTip C18 (Millipore, USA) following the manufacturer's instructions. Samples were eluted in an α-Cyano-4-hydroxycinnamic acid matrix (HCCA matrix; Bruker Daltonics, USA) dissolved in TA50 (50:50 [v/v] acetonitrile:0.1% trifluoroacetic acid, TFA). Two microliters of the eluate were spotted on AnchorChip (Bruker Daltonics) and air-dried. The spectra were obtained with MALDI-TOF Microflex-LRF mass-spectrometer (Bruker-Daltonics). Peptide calibration standard II (Bruker-Daltonics) was used as a calibrant. The acquisition was performed in flexControl v3.4 (Burker-Daltonics) in reflectron-positive mode at a laser frequency of 35 Hz (100 shots). Mass was analyzed in flexAnalysis v3.4 software (Bruker Daltonics).

Supplementary Method S9

Confirmation of the presence of disulfide bond in tagged peptides. First, to block free thiols present in the sequence, 1 nM of tagged peptides in PBS (pH 7.4) was incubated with 10-fold molar excess of 100 nM of *N*-ethylmaleimide (NEM; Sigma) for 2 h at room temperature. After incubation, the excess of NEM from the protein was removed by desalting using an *in-house* prepared Sephadex G-25 (GE Healthcare) spin column with a 1,000 – 5,000 molecular weight cut-off. Considering that the molecules containing disulfide bonds cannot be directly labeled with a maleimide, the disulfide bond in the sequence was cleaved with 500 mM of tris(2-carboxyethyl)phosphine (TCEP; Sigma) for 20 min at room temperature (reduced sample). A sample without the addition of the reducing agent was used as an oxidized control. After reduction, desalting of protein using an *in-house* prepared Sephadex G-25 (GE Healthcare) spin column was performed since the excess of reducing agent may also react with the maleimide group and, therefore, may produce false-positive results. Finally, 2 µL (2-fold molar excess) of 10 nM IRDye 800CW Maleimide (LI-COR Biosciences) were added to both samples (reduced and oxidized) and incubated for 1 h at room temperature in dark. After incubation, proteins were separated on LDS-PAGE with minor modifications (**Supplementary Method S10**).

Supplementary Method S10

LDS-PAGE of IRDye 800CW Maleimide-labeled proteins. Samples (reduced and oxidized) after incubation with IRDye 800CW Maleimide were mixed with 4X LDS sample buffer (Invitrogen) as per manufacturer's instructions without heating. Electrophoresis was carried out as described in **Supplementary Method S7**. Native gel without Coomassie staining was scanned on the Odyssey CLx imaging system (LI-COR Biosciences).

Supplementary Method S11

Confirmation of the binding of 7-mer cyclic and 12-mer linear peptides to rDIII by ELISA. The 7-mer cyclic and 12-mer linear peptides were covalently bound in CovaLink NH microwell plates overnight at 4°C as per the manufacturer´s instructions (Thermo Fischer Scientific). Unbound peptides were washed with PBS (pH 7.4) and non-specific sites were blocked with 0.5% BSA in PBS for 1 h at room temperature. After blocking, wells were washed 2 times for 2 min with CovaBuffer (2 M NaCl, 40 mM $MgSO_4.7H_2O$, 0.05% Tween 20, PBS; pH 7.2). One microgram of rDIII diluted in 0.05% PBST was added in separate wells and incubated for 1 h at room temperature with constant shaking. Wells were washed 3 times for 2 min with CovaBuffer and interaction was detected with HisProbe-HRP conjugate (1:5,000 dilution in 0.1% BSA in PBST; Thermo Fisher Scientific) for 30 min at room temperature with constant shaking. After 6 washings, the chromogenic reaction was developed using a 1-Step Ultra TMB-ELISA substrate (Thermo Fisher Scientific). After 25 min, the reaction was stopped with 2 M H_2SO_4 and absorbance was measured at 450 nm. For negative controls, either rDIII or peptides were excluded from the protocol. The assay was performed in triplicates.

Supplementary Method S12

Blocking of interaction between rDIII and proteins of hBMECs by peptides (ELISA). First, different concentrations of rDIII were tested to get the minimum concentration of rDIII required to show the interaction with hBMECs proteins. Protein extract of hBMECs (4 µg/well) diluted in coating buffer (0.5 M Na_2CO_3, 0.5 M $NaHCO_3$; pH 9.5) was incubated in the microtiter wells for overnight at 4°C. Unbound hBMECs proteins were washed with PBS (pH 7.3) containing 0.05% Tween 20 (0.05% PBST) and non-specific binding sites were blocked with 0.5% BSA in 0.05% PBST. rDIII at different concentrations (5 µg, 2.5 µg, 2 µg, 1 µg, 500

ng, 250 ng, 125 ng, and 62.5 ng) diluted in 0.05% PBST was added into wells and incubated for 3 h at room temperature and constant shaking. Wells were washed 3 times with 0.05% PBST and incubated with HisProbe-HRP conjugate (1:5,000 dilution in 0.1% BSA in 0.05% PBST; Thermo Fisher Scientific) for 30 min at room temperature and constant shaking. After the rigorous washings with 0.05% PBST, the chromogenic reaction was developed using a 1-Step Ultra TMB-ELISA substrate (Thermo Fisher Scientific). The reaction was stopped after 25 min with 2 M H_2SO_4 and the absorbance was measured at 450 nm. For negative control, rDIII was excluded from the experiment.

To block the interaction, hBMECs proteins (4 µg/well) were coated into the microtiter wells of the ELISA plate. The wells were washed and non-specific binding sites were blocked as described above. Simultaneously, 2 µg of rDIII (0.15 nM) were pre-incubated with 2.2 µg of each peptide (10-fold molar excess, 1.5 nM; diluted in PBS) for 1 h at room temperature. After blocking, the wells were washed 3 times with 0.05% PBST, pre-incubated rDIII was added and incubated for 3 h at room temperature with constant shaking. Wells were washed 3 times with 0.05% PBST and incubated with HisProbe-HRP conjugate (Thermo Fisher Scientific) as described above. The chromogenic reaction was developed using a 1-Step Ultra TMB-ELISA substrate (Thermo Fisher Scientific). The reaction was stopped after 25 min with 2 M H_2SO_4 and the absorbance was measured at 450 nm. hBMECs proteins incubated with untreated rDIII were used as a positive control. For negative control, rDIII was excluded from the experiment. The assay was performed in triplicates.

Supplementary Method S13

Blocking of interaction between rDIII and proteins of hBMECs by peptides (Western blot).

Two hundred micrograms of proteins extracted from hBMECs were separated on 2-well PAGE

gel (12% polyacrylamide gel) and transferred on 0.45 µm nitrocellulose membrane (Amersham Protran; GE Healthcare) for 1 h at 30 V. Details of LDS-PAGE are described in **Supplementary Methods S7**. The membrane was cut horizontally to obtain proteins of molecular weight between ~10 kDa to ~25 kDa. Subsequently, this membrane was sliced vertically to obtain 0.3 cm strips, which were then used in Western blot (**Supplementary Fig. S8**).

The NC strips with transblotted hBMECs proteins were first incubated with rDIII at various concentrations to find out the minimal concentration of rDIII required to detect the interaction with hBMECs proteins. Non-specific binding sites were blocked with 1% BSA in 0.05% TBST (pH 7.2) for 1 h at room temperature and constant shaking followed by a washing step for 5 min with 0.05% TBST. The NC strips were incubated with rDIII at different concentrations (5 µg, 2.5 µg, 2 µg, 1 µg, 500 ng) diluted in 1 mL of 0.05% TBST for 3 h at room temperature and constant shaking. After 3 washings with 0.05% TBST, strips were incubated with HisProbe-HRP conjugate (1:5,000 dilution in 0.1% BSA in 0.05% TBST; Thermo Fisher Scientific) for 30 min at room temperature. Six washings, each of 5 min were performed before incubating the strips in SuperSignal chemiluminescence substrate (Thermo Fisher Scientific). The signal was captured on the C-DiGit Blot Scanner (Odyssey CLx, LI-COR Biosciences). For negative control, rDIII was excluded from the above-mentioned protocol.

To block the interaction, the NC strips were blocked as described above. Simultaneously, 2.5 µg (0.19 nM) of rDIII were pre-incubated with 2.75 µg of each peptide (10-fold molar excess; 1.9 nM) in TBS for 1 h at room temperature. Further on, strips were incubated with pre-incubated rDIII for 3 h at room temperature with constant shaking. After 3 washings with 0.05% TBST, strips were incubated with HisProbe-HRP conjugate as described above. After washings, the strips were incubated in SuperSignal chemiluminescence substrate

(Thermo Fisher Scientific) and the signal was captured on the C-DiGit Blot Scanner (Odyssey CLx, LI-COR Biosciences). Incubation of the NC strip with untreated rDIII was used as a positive control. For negative control, rDIII was excluded from the above-mentioned protocol.

Supplementary Method S14

Blocking of adhesion of rDIII on cultured hBMECs by peptides (immunocytochemistry). First, different concentrations of rDIII were tested to get the minimum concentration of rDIII required to detect the adhesion of the domain to the cultured hBMECs. In short, hBMECs were cultured on collagen type I (Sigma) coated coverslips in a 12-well culture plate in EBM-2 medium (Lonza) as was described in **Supplementary Method S1** until 70% confluency. Cells were washed 2 times with PBS (pH 7.4) and fixed with acetone/ethanol (8:2 [v/v]) for 10 min at 4°C. Cells were washed 3 times with PBS (pH 7.4) and incubated with rDIII at different concentrations (20 μg, 15 μg, 10 μg, 5 μg, 2.5 μg, 2 μg, and 1 μg) diluted in 1 mL of PBS (pH 7.4) for 1 h at room temperature with gentle shaking. After 3 washings with PBS (pH 7.4), cells were incubated with anti-6x His antibody conjugated with FITC (1:500 dilution in 0.05% PBST with 1% BSA; Abcam, UK) overnight in dark at 4°C with gentle shaking. After 4 washings (5 min each) with 0.05% PBST (pH 7.4) and 1 with PBS, coverslips were taken out of the wells, dipped in ethanol for 2 - 3 sec, and mounted using Fluoroshield with DAPI (Sigma). Scanning was performed on LSM-710 microscope (Zeiss, Germany) using 359 – 461 nm filter for DAPI and 495 – 519 nm filter for FITC. The assay was performed in biological triplicates. In the case of the negative control, rDIII was excluded from the experiment.

To block the adhesion, hBMECs were cultured and fixed as described above. Simultaneously, 5 μg (0.39 nM) of rDIII pre-incubated with 10-fold molar excess (3.9 nM; 5.5 μg) of each peptide (pre-incubation was performed as described in **Supplementary Methods**

S12 and **Supplementary Methods S13**) diluted in 1 mL of PBS for 1 h at room temperature with gentle shaking. Cells were incubated with anti-6x His antibody conjugated with FITC and mounted with Fluoroshield with DAPI (Sigma) as described above. Scanning was performed as mentioned above. The assay was performed in biological triplicates. As a positive control, untreated rDIII (5 µg diluted in 1 mL of PBS) was used in the assay. In the case of the negative control, rDIII was excluded from the experiment.

Supplementary Method S15

Cytotoxicity assay. hBMECs were cultured in 96-well TPP tissue culture plates (Sigma) in supplemented EBM-2 medium (Lonza) to obtain ~70% confluency (as described in **Supplementary Method S1**). The peptides in concentrations of 1 µM, 2 µM, 4 µM, and 6 µM (diluted in 100 µL of EBM-2 medium supplemented only with 10% FBS, 10 mM HEPES and 1 ng/mL bFGF; Sigma) were added and incubated for 24 h at 37°C in a humid atmosphere of 5% CO_2. Untreated cells served as a negative control (100% viability). For positive control, cells treated with 0.01% Triton X-100 were used. After incubation, the mixture of XTT and activation reagent (5:0.1 [v/v], 50 µL each well) was added to each well and incubated for an additional 3 h at 37°C in a humid atmosphere of 5% CO_2. The absorbance was measured at 450 nm. The assay was performed in replicates. The viability (%) of endothelial cells was calculated using the formula: Cell viability (%) = $[(A_{sample} - \bar{x}A_{blank}) \times 100] / (\bar{x}A_{negative\ control} - \bar{x}A_{blank})$

Supplementary Method S16

Assessment of the hemolytic activity. Sheep whole blood (10 mL) was centrifuged at 2000 rpm for 5 min at room temperature. The red blood cells (RBCs) were carefully separated from

plasma and washed 3 times with saline (0.9% NaCl). The washed RBCs were resuspended in saline (50 mL). Peptides at two different concentrations (1 µM and 6 µM) resuspended in saline solution (100 µL) were added to the RBCs suspension (100 µL). As a positive control (100% lysis), 0.1% Triton X-100 was used. Negative control (0% lysis) was prepared by adding only saline (100 µL) to the RBCs suspension (100 µL). All samples were incubated at 37°C for 1 h and 5 h. The mixtures were gently shaked every 30 min. After incubation, the RBCs were separated by centrifugation (2000 rpm for 5 min) and the supernatant (100 µL) was incubated for 30 min at ambient conditions to oxidize the hemoglobin. The absorbance of oxyhemoglobin present in the supernatant was assessed by absorption at 414 nm. The assay was performed in triplicates. The hemolysis (%) of the RBCs was calculated using the following formula: Hemolysis (%) = $[(A_{\text{sample}} - \bar{x}A_{\text{negative control}}) / \bar{x}A_{\text{positive control}}] \times 100$.

Supplementary Method S17

Assessment of the ability of peptide to block infection in cultured cells

The ability to block infection in cultured cells was assessed using virus like particles (VLP) that possess C, M and E proteins, and carry luciferase reporter gene (*Fluc* gene). The custom synthesized VLP (Axon neuroscience, Slovakia) was first subjected to virus titration ($TCID_{50}$/ml) exactly as described before[1]. First, the HEK293T cells (kind gift from Axon Neuroscience) were seeded in 96 well cell culture plate (20,000 cells/well, TPP 96 well cell culture plate, Thermo Scientific) in 150 µl of the DMEM medium with L-glutamin and high glucose (Gibco, Thermo Scientific) containing 10% (vol/vol) FBS, 2% (vol/vol) HEPES and 1% (vol/vol) penicillin–streptomycin. Cells were grown overnight and the medium was discarded just before addition of the VLPs. The VLPs were first diluted 10-fold followed by threefold serial dilutions as shown in **Supplementary Fig. S17** in separate 96 well plate (designated as

dilution plate). Each dilution had 6 replicates. All VLP dilutions were made in DMEM medium without addition of the antibiotics. Diluted VLPs were transferred to the cell culture plate. Six wells were maintained without addition of the VLPs as cell control. After incubation at 37°C and 5% CO_2 for 24 h, the cells were lysed with 20 µl lysis buffer provided in the luciferase assay system kit (Promega, USA) for 5 min at room temperature. The lysate was transferred to white 96 well plate (luminescence compatible), 100 µl of the luciferase assay reagent provided in the kit was added and the luminescence was measured immediately on Cytation 7 (Biotek, USA) instrument with following parameters: integration time – 10 sec, read height 5.4 mm and the gain 240. Values obtained from Cytation 7 were pasted in the excel template provided in **Supplementary dataset 1.** Please note that, this template was adopted from the the recently published article [1] and the calculation of $TCID_{50}$/ml was performed according to Reed-Muench method[2].

To measure the ability to block the infection, peptides were serially diluted as presented in **Supplementary Fig. S18,** which then mixed with VLP (400-500 $TCID_{50}$/ml) and incubated for 90 min. After incubation, solutions from each well were transferred to the cell culture plate and incubated at 37°C and 5% CO_2 for 24 h. After incubaton, suparnatnat was removed carefully from each well, 20 µl lysis buffer provided in the luciferase assay system kit (Promega, USA) was added and plate was incubated for 5 min at room temperature. The lysate was transferred to white 96 well plate (luminescence compatible), 100 µl of the luciferase assay reagent provided in the kit was added and the luminescence was measured immediately on Cytation 7 (Biotek, USA) instrument with following parameters: integration time – 10 sec, read height 5.4 mm and the gain 240. The amount of VLP entering the target cells was calculated by detecting the expression of luciferase, which then used to measure the neutralizing ability of the peptides, expressed in half maximal effective concentration

(EC_{50}). To calculate $EC_{50,}$ values obtained from Cytation 7 were pasted in the excel template provided in **Supplementary dataset 2** (template adopted from recently published article[1]) and calculation of EC_{50} was performed according to Reed-Muench method[2].

EC_{50} value were used to calculate half maximal effective concentration (nanograms) of papetide required to neutralize the virus using nonlinear regression (curve fit). A curve was plotted using dilution of nanobody on Y axis and amount of peptid/well on X axis as shown in **Supplementary dataset 2**. To interpolate unknowns from standard curve a sigmoidal (sigmoid, 4PL, x is concentration) model was used in Prism 8 (www.graphpad.com).

References

1 Nie, J. *et al.* Quantification of SARS-CoV-2 neutralizing antibody by a pseudotyped virus-based assay. *Nat Protoc* **15**, 3699-3715, doi:10.1038/s41596-020-0394-5 (2020).

2 Matumoto, M. A note on some points of calculation method of LD50 by Reed and Muench. *Jpn J Exp Med* **20**, 175-179 (1949).
